# Combining host and vector data informs emergence and potential impact of an Usutu virus outbreak in UK wild birds

**DOI:** 10.1038/s41598-022-13258-2

**Published:** 2022-06-18

**Authors:** Becki Lawson, Robert A. Robinson, Andrew G. Briscoe, Andrew A. Cunningham, Anthony R. Fooks, Joseph P. Heaver, Luis M. Hernández-Triana, Shinto K. John, Nicholas Johnson, Colin Johnston, Fabian Z. X. Lean, Shaheed K. Macgregor, Nicholas J. Masters, Fiona McCracken, Lorraine M. McElhinney, Jolyon M. Medlock, Paul Pearce-Kelly, Katharina Seilern-Moy, Simon Spiro, Alexander G. C. Vaux, Arran J. Folly

**Affiliations:** 1 Institute of Zoology, Zoological Society of London, Regent’s Park, London, NW1 4RY UK; 2 British Trust for Ornithology, The Nunnery, Thetford, Norfolk, IP24 2PU UK; 3 Natural History Museum, Cromwell Road, South Kensington, London, SW7 5BD UK; 4 Animal and Plant Health Agency, Woodham Lane, Addlestone, Surrey KT15 3NB UK; 5 Medical Entomology and Zoonoses Ecology group, UK Health Security Agency, Porton Down, Salisbury, SP4 0JG UK; 6 Zoological Society of London, Regent’s Park, London, NW1 4RY UK

**Keywords:** Ecology, Ecological epidemiology, Infectious-disease diagnostics

## Abstract

Following the first detection in the United Kingdom of Usutu virus (USUV) in wild birds in 2020, we undertook a multidisciplinary investigation that combined screening host and vector populations with interrogation of national citizen science monitoring datasets to assess the potential for population impacts on avian hosts. Pathological findings from six USUV-positive wild passerines were non-specific, highlighting the need for molecular and immunohistochemical examinations to confirm infection. Mosquito surveillance at the index site identified USUV RNA in *Culex pipiens s.l.* following the outbreak*.* Although the Eurasian blackbird (*Turdus merula*) is most frequently impacted by USUV in Europe, national syndromic surveillance failed to detect any increase in occurrence of clinical signs consistent with USUV infection in this species. Furthermore, there was no increase in recoveries of dead blackbirds marked by the national ringing scheme. However, there was regional clustering of blackbird disease incident reports centred near the index site in 2020 and a contemporaneous marked reduction in the frequency with which blackbirds were recorded in gardens in this area, consistent with a hypothesis of disease-mediated population decline. Combining results from multidisciplinary schemes, as we have done, in real-time offers a model for the detection and impact assessment of future disease emergence events.

## Introduction

Usutu virus (USUV; family: *Flaviviridae*, genus *Flavivirus*) is a zoonotic single-stranded RNA virus, first detected in Africa that has spread to multiple countries across mainland Europe since 1996, where there are currently ten recognised lineages of USUV co-circulating^1,2^. USUV has an enzootic cycle with transmission between mosquito vectors and birds as the main amplifying hosts^3^. Whilst a variety of predominantly passerine hosts are susceptible, epizootic mortality of wild birds due to USUV has principally affected Eurasian blackbirds (*Turdus merula*)^4^ and on a scale sufficient to cause population decline in some countries (e.g. Germany^5^). The blackbird is a common and widespread wild bird species in Europe; its frequent utilisation of peri-domestic garden habitats combined with USUV susceptibility make it an ideal sentinel species for USUV detection. Whilst USUV infection in humans is apparently rare, and evidence supports it being predominantly asymptomatic, small numbers of cases of disease, typically with neurological signs, have occurred in regions where USUV is established^1,6^. Therefore, surveillance for the early detection and likely extent of circulating USUV provides valuable information for public health risk assessment.

USUV lineage Africa 3.2 was detected for the first time in the United Kingdom (UK) in five blackbirds and one house sparrow (*Passer domesticus*) in Greater London at the Zoological Society London (ZSL) London Zoo (51°32′ N 0°9′ E) in August 2020^7^. However, the extent and impact of this incursion on local wildlife populations was unclear. Consequently, and more generally, there is a growing recognition of the need to develop methods to facilitate early detection of emergent disease in free-living wildlife populations and to appraise the population-scale impact of these events. Here we present a multidisciplinary investigation following the first detection of USUV in the UK^7^ which combines results from independent host and vector surveillance with wildlife population monitoring schemes. Findings from these disciplines are synthesised to identify the extent and impact of USUV emergence in UK wild birds and to inform the development of future surveillance strategies which we believe may be applicable to other emerging diseases. Molecular screening for *Plasmodium* spp. was performed to appraise factors affecting host health, since avian malaria has been described as a comorbidity with USUV infection in wild birds^4,8^. Finally, mosquito blood meal analyses were conducted to explore local host feeding to inform potential species at risk of USUV exposure and advance understanding of USUV transmission networks.

## Methods

A comprehensive methodology with supporting citations is available in the Supplementary Methods.

### Host surveillance at the index site

Pathological examinations of the six USUV-positive wild birds, that were found dead or euthanised on welfare grounds, at the index site comprised systematic post-mortem examinations with detailed parasitological, microbiological, and histological investigations, including immunohistochemical labelling for the detection of flavivirus envelope (E) antigen combined with screening for avian haemoparasites using a nested PCR. Tissues from captive bird species that also died at the index site in 2020 were subjected to USUV RT-PCR assay to identify if any other species was affected during the outbreak.

### Vector surveillance at the index site

In response to the initial detection of USUV in wild birds, intensive mosquito trapping was implemented at the index site during September 2020. Four types of mosquito traps designed to detect ornithophagic and mammalophagic feeding of both native and non-native species were deployed over four locations to assess the extent of feeding preference and to collect adult mosquitoes for molecular analysis. Following morphological species identification, RNA extracted from pooled mosquitoes was subjected to USUV RT-PCR to identify whether there was evidence of virus circulation in the local vector population. Positive RNA samples were submitted for next generation sequencing to determine the USUV lineage. Virus isolation was attempted on pooled mosquito homogenates that tested positive for USUV RNA. DNA extracted from blood-fed mosquitoes was screened using a vertebrate specific triple primer cocktail to identify host species using Sanger sequencing.

Mosquito monitoring had previously been conducted at the index site in 2015 as part of a wider vector monitoring programme. This historical dataset on vector abundance, feeding preference and potential circulating vector-borne avian haemoparasites enabled comparison of such assemblages before and after the emergence of USUV. Mosquitoes were collected from resting sites in animal enclosures and surrounding vegetation at multiple locations across ZSL London Zoo. Species identification was performed based on morphological features. Metabarcoding of pooled DNA from 96 mosquito blood meals with order-specific primers for birds and mammals targeting the NADH dehydrogenase 2 (ND2) and cytochrome oxidase subunit I (CO1) mitochondrial genes was used to identify vertebrate hosts. PCR targeting *Plasmodium* spp. and subsequent Sanger sequencing of positive amplifications, to both identify haemoparasite species and vertebrate hosts, was conducted to appraise their role as avian haemoparasite vectors.

### Host investigations at the national scale

#### Molecular surveillance for Usutu virus in wild birds

Scanning disease surveillance for wild birds is conducted in Great Britain (coverage does not include Northern Ireland) through post-mortem examinations (PME) performed by three complementary programmes: the Garden Wildlife Health (GWH) project (www.gardenwildlifehealth.org) focuses on peri-domestic species including garden birds; Animal & Plant Health Agency’s Diseases of Wildlife Scheme investigates all submitted wild bird species; and the UK Centre for Ecology and Hydrology’s Predatory Bird Monitoring Scheme focuses on raptors and owls. Brain and kidney samples from selected target species (primarily blackbird and also Strigiformes, in which mortality has been recorded sporadically in captive birds^1^) were subjected to a specific USUV RT-PCR assay over the period 2012–2019. In 2020, as a direct response to the USUV detection in the UK, enhanced USUV surveillance was employed screening all garden bird and bird of prey submissions.

#### Syndromic surveillance and utilising the ring recovery dataset

Data from two independent schemes that record wild bird mortality were reviewed to assess if there was any signal of increased blackbird mortality in summer 2020 that might indicate wider USUV circulation. Reports of morbidity and mortality in garden wildlife (including birds) were solicited from members of the public and submitted online through GWH. Disease incident reports (DIRs) were retrospectively evaluated over the available eight-year dataset, 2013–2020. Where observations of morbidity were available, garden bird DIRs were allocated to one of seven syndromic surveillance categories; avian pox; beak/plumage abnormality; generalised ill health (e.g. lethargy, fluffed-up plumage); musculoskeletal disease; nestling mortality; neurological disease and predation/trauma. Based on the experience of wild bird mortality caused by USUV in mainland Europe (e.g.^4,9,10^), the syndromic surveillance categories generalised ill health and neurological disease were considered of particular interest as a potential signal of infection. However, USUV could not be excluded as a possible underlying cause for DIRs categorised as musculoskeletal disease, trauma, predation, drowning or tick parasitism, or where blackbirds were found dead with no observed clinical signs. The composition of syndromic surveillance categories for blackbird DIRs was compared across study years over the mosquito active period (June-November inclusive) to detect if there was any variation in 2020 that might indicate USUV-associated morbidity.

Evidence for spatial clustering of DIRs that might support a regional disease outbreak was investigated using SaTScan (www.satscan.org), whilst accounting for heterogeneities in observer effort and in blackbird population density. In the same way, we categorised DIRs of house sparrow, the second species in which USUV infection was detected, as well as robin (*Erithacus rubecula*) and starling (*Sturnus vulgaris*); the latter two were used as control species due to similarities in their 1. widespread distribution, 2. frequent use of peri-domestic habitats (therefore they are often observed by the public and likely have shared potential for mosquito exposure) and 3. diet, which primarily consists of ground and soil-living invertebrates. As an independent measure of mortality, we examined the seasonal pattern of recoveries of dead ringed blackbirds for the regions of both Greater London and the adjacent East England and South-east England regions.

#### Sentinel species population monitoring

We used two independent wild bird population monitoring datasets (1. British Trust for Ornithology’s Garden BirdWatch (GBW) and 2. the British Trust for Ornithology/Joint Nature Conservation Committee/Royal Society for the Protection of Birds Breeding Bird Survey (BBS)) to investigate blackbird population trends since 2011, when USUV was first detected as a cause of wild bird mortality in coastal Western European countries^3^. Mainland Europe is considered the likely origin of USUV in the UK^7^, therefore incursion and any associated blackbird population decline which could be attributed to USUV infection would be predicted to have occurred over the past decade since the first detection of the virus in countries with close spatial proximity (i.e. coastlines neighbouring the North Sea and English Channel^1^). BBS population indices for blackbird (collected across habitats using line transect surveys of a stratified random selection of sites) were explored for the UK and the same three regions of southern England used for the ring recovery analysis.

Measures of population change following the USUV outbreak in 2020 across all habitats are not available, therefore we calculated the mean weekly reporting rate (i.e. proportion of gardens that submitted a record in that week in which a species was reported) for blackbirds visiting a (self-selecting) sample of gardens contributing to GBW in the UK and in each of the three English regions used above. We did this for the period 2011–2019 inclusive and compared the results with those for 2020 for blackbird, house sparrow, robin and starling. Finally, we summarised the weekly maximum blackbird counts during the period 2003–2020 inclusive for Greater London alone.

## Results

### Host surveillance at the index site

Results of diagnostic testing from the six USUV-positive wild birds from the index site (2020) were reviewed to appraise the significance of USUV in terms of its contribution to cause of death and to identify comorbidities such as *Plasmodium* sp. infection. Gross lesions were non-specific and inconsistent across birds, with evidence of dehydration in four blackbirds, mild hepatosplenomegaly in one blackbird and diffuse tan discolouration of the kidneys in the house sparrow (Supplementary Table 1). Gastrointestinal and/or respiratory parasites were present in three blackbirds, and numerous coccidian oocysts were detected in the small intestinal contents of the house sparrow.

**Figure 1:**
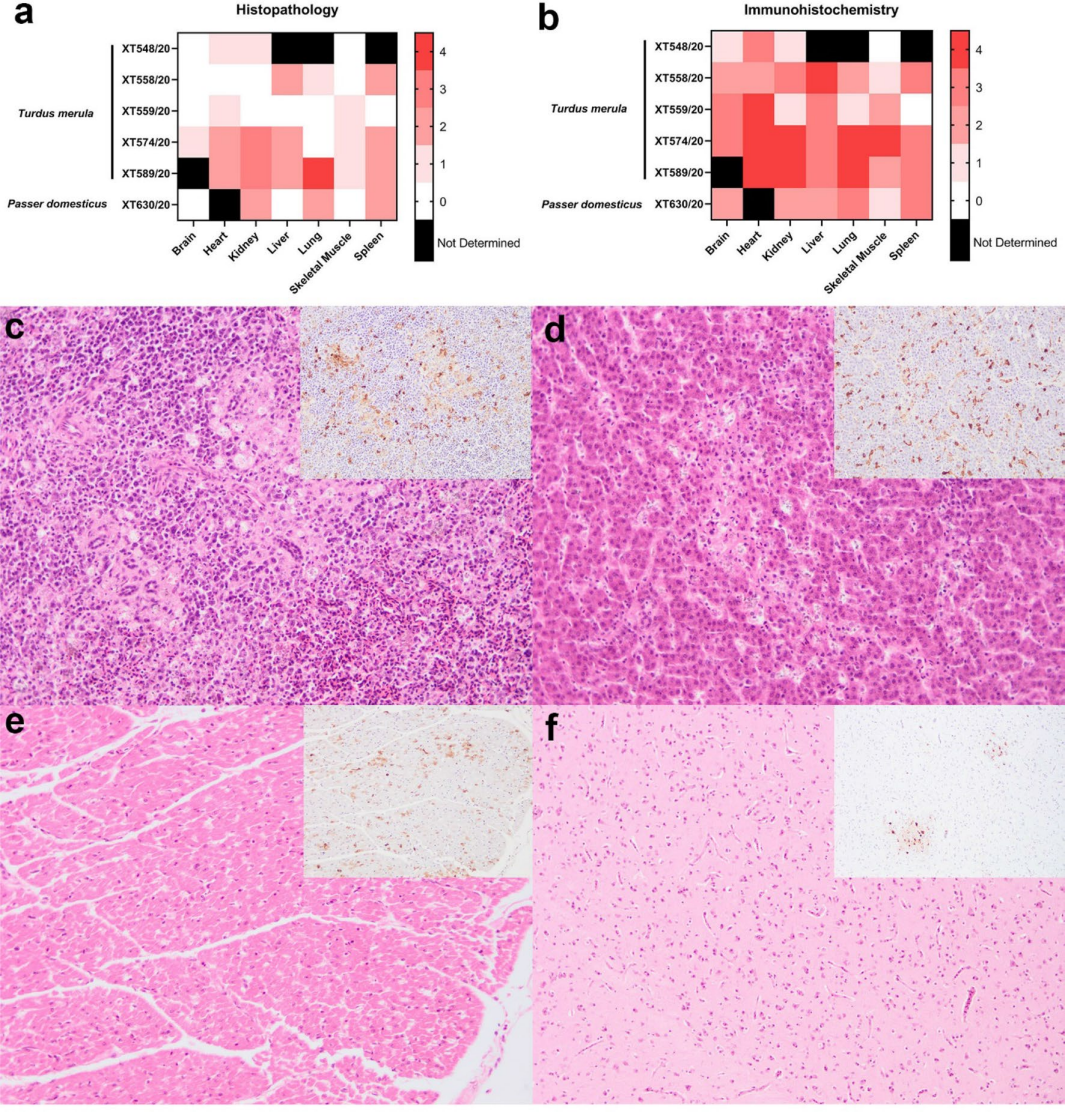
Histopathological and immunohistochemical findings of Eurasian blackbirds (*Turdus merula*) and house sparrow (*Passer domesticus*). Categorical heat maps indicate the maximum severity of histopathological changes (**a**) and the level of immunohistochemical labelling against flavivirus envelope (**b**). For histopathology 0 absent; 1 minimal; 2 mild; 3 moderate; 4 marked; for immunohistochemistry: 0 absent; 1 rare; 2 scattered; 3 confluent; 4 abundant. No histopathological findings were considered marked. Black indicates where no samples were available for analysis. Representative histopathology and immunohistochemical sections of tissues from Usutu virus infected blackbird (**c–f**). Virus antigens are associated with areas of necrosis in the spleen (**c**) and liver (**d**) but also present in areas without histological changes. Minimal histological changes in the heart (**e**) and brain (**f**) but with moderate and scattered amount of virus antigens in respective tissues. Serial sections are stained with haematoxylin and eosin and flavivirus anti-envelope antibody (insets). Original magnification 200x. Histology scores: 2 (spleen, liver, heart), brain (1). IHC scores: 3 (spleen, liver), 4 (heart), 2 (brain).

Microscopic lesions were similarly non-specific and inconsistent, with spotty necrotising hepatitis (3/5)^11^, lymphohistiocytic interstitial nephritis (3/5) and necrotising splenitis (3/5) being the most common diagnoses in the five blackbirds, with the latter two lesions also present in the house sparrow; these lesions varied in severity from minimal to moderate in both species (Fig. 1). Brain was examined from four blackbirds with mild focal necrosis observed in one case only. Immunohistochemical labelling of flavivirus envelope revealed antigen in a wide variety of organs, including brain, even where histological changes were minimal or absent (Fig. 1). Whilst *Plasmodium* spp. DNA (Supplementary Table 1) was detected using PCR in two of the blackbirds, no schizonts indicative of avian malaria were observed on microscopical examination of tissues, therefore this infection was considered likely to be an incidental finding.

### Vector surveillance at the index site

Metabarcoding of 96 blood-fed *Cx. pipiens* s.l. collected in 2015 identified ornithophagic but no mammalophagic behaviour. Wild bird hosts comprised predominantly blackbird, along with starling, robin and blue tit (*Cyanistes caeruleus*) (Supplementary Worksheet 1–3). Feeding on a variety of captive bird hosts across six orders was also detected. *Plasmodium* spp. were detected in 12/96 mosquitoes (Supplementary Worksheet 4), of which 10 had fed on blackbirds, one on Abdim’s stork (*Ciconia abdimii*), and one bloodmeal for which the host could not be identified. Four of 11 blood-fed *Cx. pipiens* s.l. specimens collected in September 2020 produced sufficient sequence to identify a vertebrate host. In each case the sequence obtained shared > 98.5% identity with a voucher specimen of Abdim’s stork (Genbank accession number KU722448).

During September 2020, a total of 489 mosquitoes were collected during 266 trap nights over 26 nights (Supplementary Table 2). Almost all mosquitoes were *Cx. pipiens* s.l. (n = 468 female, 16 male) with a small number of *Culiseta annulata* (4 female, 1 male) (n = 5). *Cx. pipiens* s.l. samples were pooled and tested for presence of USUV RNA. Thirteen percent (6/46) of *Cx. pipiens* s.l. pools tested RT-PCR positive for USUV RNA (Supplementary Table 3; Supplementary Figure 1). Whilst a complete genome was not recovered from any of the USUV positive pools, reads from each sample were mapped to the Africa 3.2 Greater London USUV genome (Genbank accession number MW001216), providing varying levels of genome coverage which supported detection of USUV RNA from four pools. The remaining two RT-PCR positive pools had high Ct values (cycle threshold > 35) combined with a low number of mapped reads, and were therefore considered to be ambiguous test results. No virus was isolated through two rounds of cell culture from any of the *Cx. pipiens* s.l. pools which tested positive by RT-PCR for USUV RNA. Five *Cs. annulata* mosquitoes were extracted individually and each tested USUV RT-PCR negative.

### Host investigations at the national scale

#### Molecular surveillance for Usutu virus in wild birds

Over the period 2012–2019 inclusive, samples from 372 wild birds (including 43 blackbirds) tested negative for USUV RNA (Supplementary Table 4). Samples from a total of 115 wild birds (23 species, 16 families from seven orders) were tested for USUV RNA by RT-PCR in 2020 (Supplementary Table 5a). The submissions included fifteen blackbirds (from 10 sites in five English regions and Wales) and seven house sparrows (from four sites in four English regions). Apart from the five blackbirds and single house sparrow reported previously^7^, all wild birds tested in 2020 were negative for USUV RNA.

Samples from a total of 19 captive birds from ZSL London Zoo (16 species, 13 families from 7 orders) that were examined post-mortem over the period 17th February to 17th November 2020 were submitted for USUV RT-PCR and all tested negative (Supplementary Table 5b). PMEs supported an alternative cause of death in all captive birds, with no unexplained mortality or lesions suggestive of USUV infection (e.g. hepatosplenomegaly^4^).

#### Syndromic surveillance and utilising the ring recovery dataset

**Figure 2:**
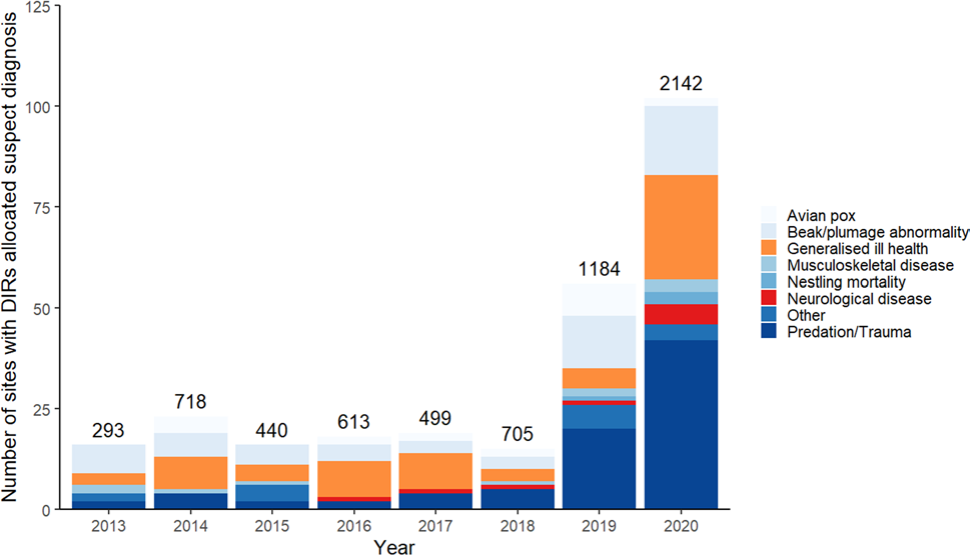
Number of sites from which Eurasian blackbird (*Turdus merula*) disease incident reports (DIRs) were submitted in Great Britain by syndromic disease surveillance category, June–November inclusive, 2013–2020 (n = 265); improved reporting was implemented in 2019*. Data labels indicate total number of sites from which passerine DIRs were submitted in respective years. *Whilst the number of blackbird morbidity reports was relatively consistent across the period 2013–2018 inclusive, a marked increase in the absolute number of DIRs occurred in 2019, which was reflected across the Passeriformes species, and for hedgehogs as a control group (Supplementary Fig. 2). This increase can be explained by a change in the online platform which prompted an increased number of reports made by participants in the GBW scheme. A further increase in the scale of reporting occurred in 2020, again across garden wildlife species, is accounted for by the COVID-19 pandemic, with more people working from home and observing wildlife in their gardens and immediate surrounding areas.

Blackbird morbidity and/or mortality was reported to GWH from 303 sites (Fig. 2) from across Great Britain during the active mosquito seasons (June-November) over the period 2013–2020 inclusive: 228 of 368 (62%) reports were of dead birds. Of these, the majority (88%; 201/228) involved a single blackbird, whilst the remaining 12% (27/228) comprised an average of 2.7 birds (range 2–7). For the multiple mortality reports, no morbidity was observed in 37% (10/27), with generalised ill health (26%; 7/27) and predation/trauma (22%; 6/27) the most frequently allocated syndromic surveillance categories where illness had been observed. USUV, as an underlying cause, could not be excluded in 78% (286/368) of reports.

The distribution of blackbird DIRs by reporting category differed in 2020 compared to the average of the preceding seven years for the period June-November inclusive (*χ*^2^_7_ = 19.9, *p* < 0.001) and when compared with 2019 alone (χ^2^_7_ = 18.6, p < 0.001) (Supplementary Table 6). This, though, was caused by a lack of avian pox records in 2020; the relative number of DIRs in the generalised ill health and neurological disease categories for the same six-month period in 2020 was similar to previous years (mean 32%, range 11–56% for 2013–2019 and 30% in 2020).

**Figure 3:**
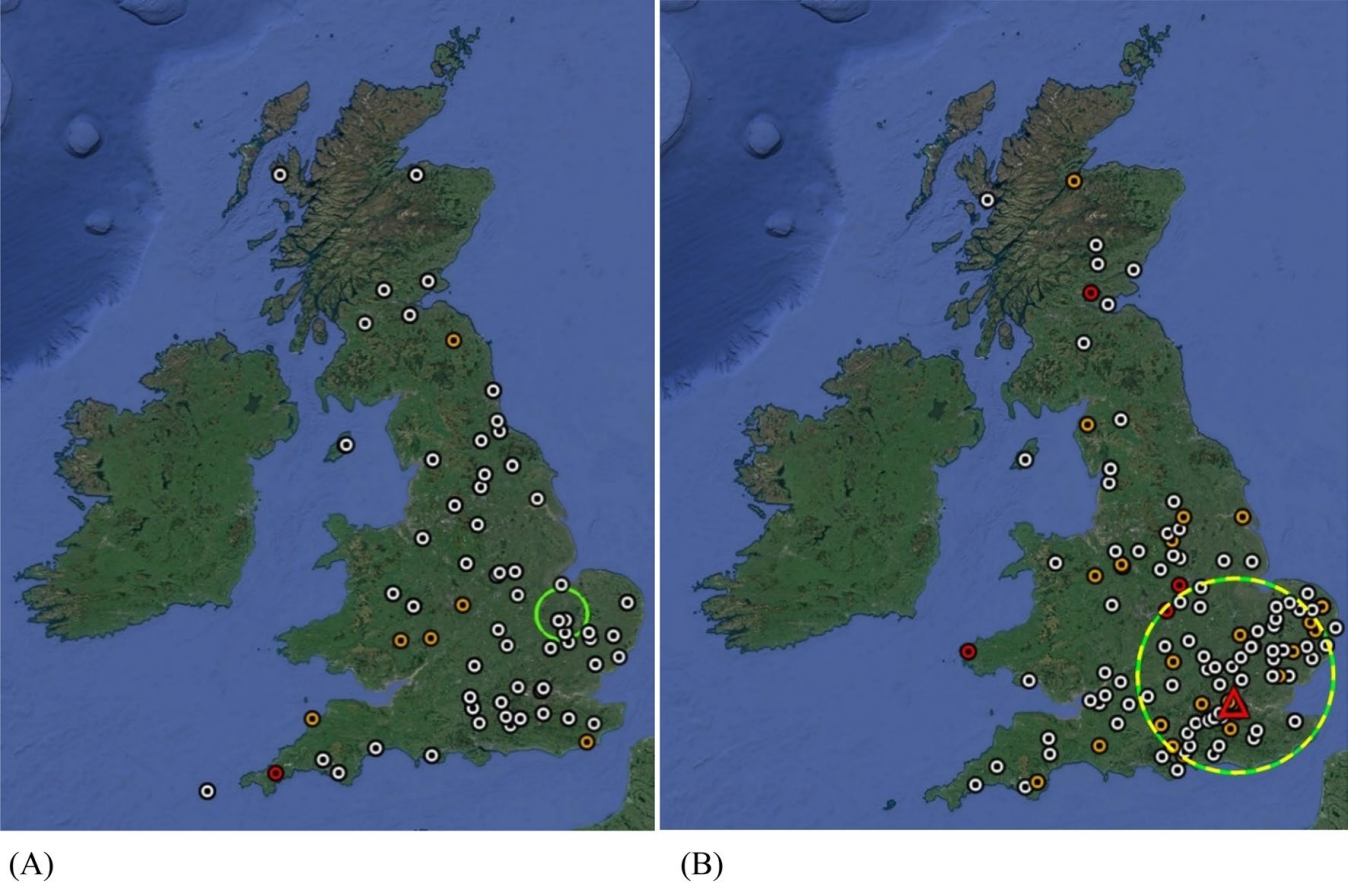
Distribution of disease incident reports (DIRs) in Eurasian blackbirds (*Turdus merula*) from Great Britain, June -November inclusive, 2019 (**A**; n = 56) and 2020 (**B**; n = 120). Red circles represent DIRs consistent with neurological disease; orange circles represent DIRs involving blackbirds exhibiting signs of generalised ill health; white circles represent all other morbidity and/or mortality DIRs (removing categories where USUV could reasonably be excluded as the cause i.e. avian pox, nestling mortality, beak and plumage abnormality); red triangle denotes site of confirmed USUV infection. Green circle (**A**, 2019) indicates statistically significant cluster of reports, accounting for heterogeneity in blackbird population density. Hatched circle (**B**, 2020) indicates statistically significant cluster of reports, accounting for spatial heterogeneity in surveillance effort by using number of sites from which passerine DIRs were submitted in each county (yellow) and for heterogeneity in blackbird population density (green); clusters are of identical size and location. Map created with Google Earth Pro. Version 7.3.3.7699 (2020) (https://www.google.com/intl/en_uk/earth/versions/#earth-pro).

There was no evidence of spatial clustering of blackbird multiple mortality incidents, or DIRs of generalised ill health and neurological disease categories combined, in 2019 or 2020 (Fig. 3a). However, a significant spatial cluster (127 km radius) of all blackbird DIRs (excluding the three categories not consistent with USUV) was identified in the Greater London, South East and East of England region (centred circa 32 km from the index site) in 2020 (Fig. 3b). This cluster was of identical size and location when heterogeneity in reporting rate was accounted for either by the total number of sites from which passerine DIRs were submitted (log likelihood ratio (LL) = 9.3; relative risk (RR) = 2.2; p = 0.01), or by the mean blackbird population density (LL = 35.0; RR = 4.8; p < 0.001). No similar consistent pattern was observed for house sparrow (Supplementary Figure 3a): The available dataset of DIRs for robin and starling had ≤ 50 DIRs per season, therefore formal spatial analyses were not conducted (Supplementary Figure 3b–c).

During 2020, the number of reports of dead ringed blackbirds was reduced in the first half of the year, but similar to that of previous years in the second half of the year (Supplementary Figure 4), suggesting no increase in the scale of blackbird mortality over the period of USUV detection.

#### Sentinel species population monitoring

**Figure 4:**
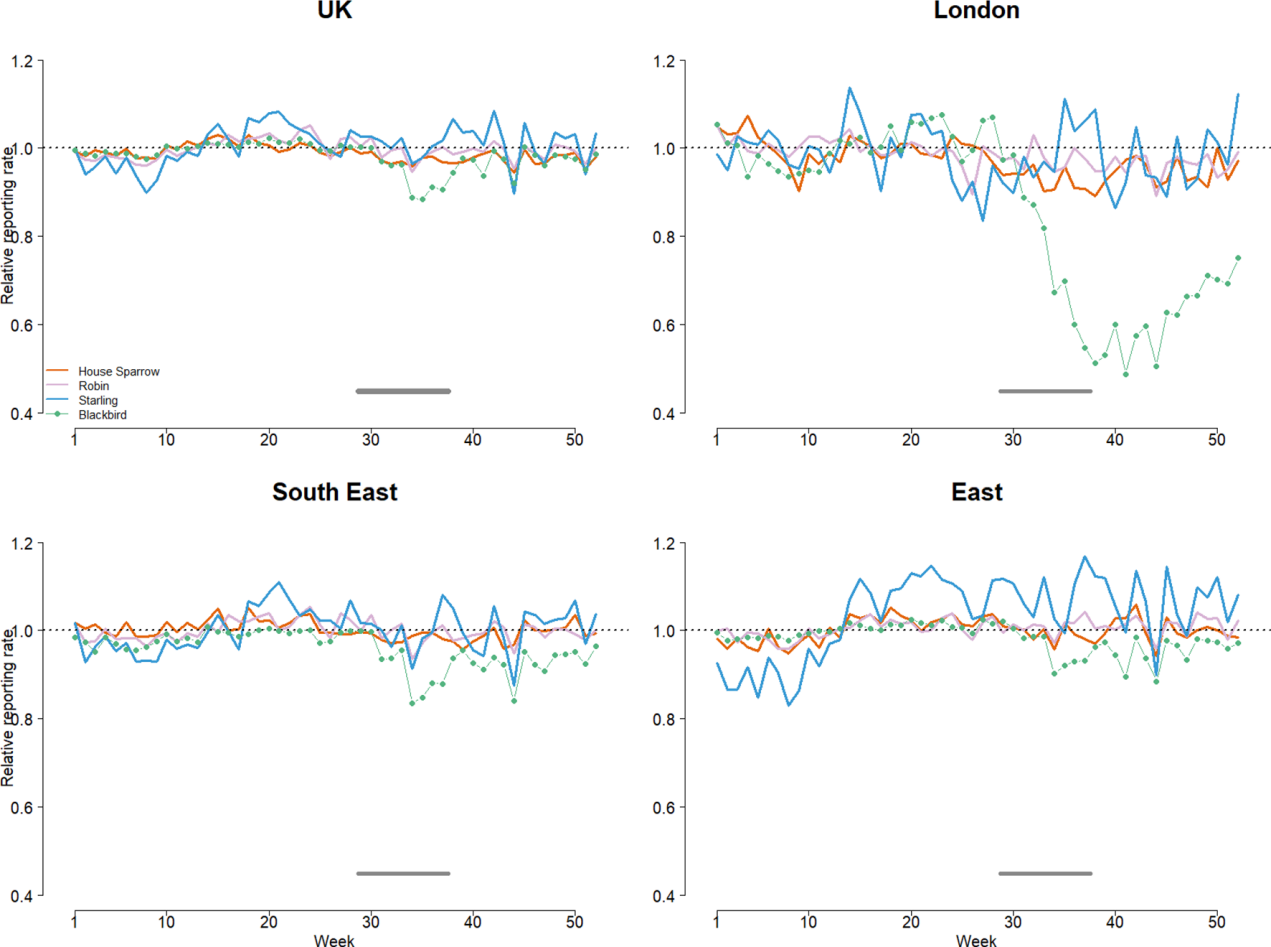
Relative weekly reporting rate in 2020 for Eurasian blackbird (*Turdus merula*), house sparrow (*Passer domesticus*), robin (*Erithacus rubecula*) and starling (*Sturnus vulgaris*) throughout the year. Data from British Trust for Ornithology’s Garden BirdWatch survey. Weeks are numbered from 1 (1st week of January) through 52 (last week of December) and a relative reporting rate of 1 indicates the presence of a species in a similar proportion of gardens in 2020 compared to the 2011–2019 average. The grey horizontal line represents the period from first USUV detection (15th July 2020) through blackbird post-mortem examination and latest detection (17th September 2020) through mosquito surveillance and molecular testing.

Between 2011 and 2019, the BBS population trends for blackbirds in the UK and South East England were stable across regions and reporting years (Supplementary Figure 5). The GBW reporting rate of blackbirds in Greater London declined by up to 50%, beginning in week 29 (mid-July) 2020 and lasting into November (week 44), contemporaneous with the period of USUV detection (Fig. 4). Whilst this value subsequently began to recover, it remained circa 32% reduced in December 2020 in contrast to the same time in 2019 (mean ± SE 2019: 0.78 ± 0.01, 2020: 0.53 ± 0.01; χ^2^_1_ = 178, p < 0.001). A much less marked reduction in blackbird reporting rate was observed over the same period in both the South East (7%) and East of England (5%) regions, with these values returning to their historical range by the end of the year. In contrast, no notable variation in house sparrow, robin or starling reporting rate was observed. A similar pattern of reduction was observed in the weekly maximum number of blackbirds recorded per garden in Greater London (Supplementary Figure 6).

## Discussion

Here we have used existing surveillance to detect an emerging wildlife disease and appraise its impact by combining traditional host and vector screening with utilisation of national datasets generated by citizen scientists. Following the detection of USUV in the UK in 2020^7^, whilst national surveillance identified no further cases of USUV infection in wild birds that year, we discovered a significant cluster of blackbird DIRs and an overlapping regional reduction in reported blackbird observations, possibly indicating disease-mediated population decline. Our investigation also identified mosquito vectors at the index site that were positive for USUV RNA, suggesting that ongoing virus transmission was likely.

The most prevalent and notable histological changes in the blackbirds and house sparrow with confirmed USUV infection were those in the liver and spleen, consisting of necrosis and lymphohistiocytic inflammation along with moderate to abundant virus antigen labelling. Whilst neurotropism resulting in brain necrosis and lymphohistiocytic inflammation has been reported in studies which examined large numbers of wild blackbirds with USUV infection in continental Europe^4,12^, we found minimal evidence of neural lesions in the five wild birds examined in this study. Although, histopathological changes in other tissues were generally non-specific, immunolabelling demonstrated widespread virus antigen distribution in both bird species, which is similar to reports of USUV infection elsewhere^4,13,14^. Immunolabelling was disproportionately greater in the brain and heart in contrast to the minimal or absent histological changes observed in these organs: similar contrasting results of histological and immunohistochemical examinations of USUV-infected wild birds have previously been reported^12^. Although only brain and kidney samples were examined using USUV RT-PCR, our findings, together with earlier reports^4,14^, demonstrate that viral antigen can be detected in abundance in the heart and liver, suggesting that these organs could be useful for molecular diagnostic sampling.

A differential for necrotising lesions in European passerines, and a comorbidity detected in blackbirds with USUV infection, is *Plasmodium* spp. infection^4,8,15^. DNA of the same *Plasmodium* spp. as detected in the tissues of USUV-positive blackbirds from the ZSL London Zoo site in 2020 was identified in *Cx. pipiens* s.l. that fed on blackbird hosts at this site previously in 2015, supporting endemic avian haemoparasite infection of this wild bird species at this location. In contrast to the results reported from USUV-positive blackbirds in the Netherlands^4^, no exo-erythrocytic stages of haemoprotozoa indicative of avian malaria were observed histologically in the two UK blackbirds positive for *Plasmodium* DNA. Since histological examination has limited sensitivity, in situ hybridisation could be used to further appraise the clinical significance of this co-infection in the future^16^.

Zoological collections are ideally placed to form part of wildlife disease surveillance networks and have already contributed to flavivirus detection in mainland Europe^10,13,17,18^. The collection grounds at ZSL London Zoo are well monitored for evidence of morbidity or mortality in synanthropic wildlife; this unusually high level of vigilance is considered the likely explanation for detection of USUV at such a location. Recent import of infected captive birds can be excluded as a potential route of USUV introduction as the COVID-19 pandemic had led to suspension of animal movements into the zoological collection. Following USUV detection in synanthropic wildlife, preemptive management practices were employed to safeguard the health of captive animals (Supplementary Materials 1); there was no evidence of USUV-associated disease in the collection animals.

The majority of mosquitoes trapped in 2020 were primarily ornithophagic *Cx. pipiens* s.l., a known vector for USUV^1^ and a common species in temperate urban habitats. This mosquito species was also the most frequently detected at the ZSL London Zoo site in 2015, during historical trapping sessions^19^ and at two zoological collections in northern England^20^. Bloodmeal analyses from mosquitoes at ZSL London Zoo in 2015 and 2020 demonstrate that this species feeds on both wild and collection birds, as would be expected for a generalist ornithophagic mosquito^21^. In addition, targeted mosquito surveillance in 2020 confirmed circulating USUV in multiple *Cx. pipiens* s.l. pools at the index site over a three-week period subsequent to the detection of USUV-associated wild bird mortality. This further demonstrates that local mosquito trapping combined with PCR screening is useful as part of an integrated surveillance programme^22^ and provides evidence that native vectors in the UK may facilitate the onward transmission of USUV to susceptible hosts following an emergence event.

Wild bird flavivirus surveillance in Great Britain integrates submissions from three schemes, each with a different taxonomic focus. These convenience samples inevitably lead to skews in species coverage. Although a common garden bird, the number of blackbirds tested for USUV was modest at 2–8 per annum over the period 2012–2019. A communication programme to raise awareness of blackbirds as a sentinel species for USUV, involving a range of stakeholder communities (e.g. non-governmental organisations, wildlife rehabilitators and veterinary surgeons) could help to increase the volume of submissions and, by extension, the ability to rapidly identify the occurrence of USUV in this species. The potential value of target species as sentinels within wild bird surveillance networks has been highlighted for other pathogens, e.g. highly pathogenic H5N1 avian influenza and West Nile virus^23,24^. In addition to this passive surveillance focused on disease detection in avian hosts, active targeted serosurveys could be conducted to identify cryptic exposure of subclinically affected birds in the future. Given the logistical challenges around active serosurveys in wild birds, screening of archived samples from captive birds in the zoological collection may provide a means to further appraise the extent of USUV circulation, as has previously been undertaken at other collections in mainland Europe^25,26^.

Local reductions of blackbird populations have been reported following USUV outbreaks in mainland Europe^27–29^, but numbers recorded by the BBS have been stable in the UK and Greater London since 2011 when USUV incursion would be predicted most likely to have occurred on the basis of spatio-temporal patterns of spread in mainland Europe^3^ until the latest data are available from 2019 (Supplementary Figure 5). Whilst our index site detection of USUV is unlikely to represent the incursion event, and earlier sporadic or localised USUV incidents prior to 2020 may have occurred^7^, based on historical blackbird population trends it seems plausible that the existing surveillance system enabled rapid detection of this emerging infectious disease.

Significant clustering of blackbird DIRs was observed in the Greater London, South East and East of England regions in 2020. These results should be interpreted with care given the potential for biases with these opportunistic data and the absence of confirmed aetiology for the DIRs, however, these findings are consistent with a regional increase in blackbird morbidity and mortality in summer 2020 around the USUV index site. Consequently, it is likely that further blackbirds, in addition to those recovered for PME, were infected and died with USUV. Whilst no evidence of an increase in generalised ill health or neurological disease category blackbird DIRs was found in 2020, particular attention should be paid to early detection of clusters of DIRs of these categories as a potential signal of USUV occurrence in the future.

One indicator, the dead bird ringed recovery dataset, did not support increased scale of blackbird mortality in Greater London; however, the dataset is small and vulnerable to variation in observer bias (e.g. related to COVID-19 induced lockdown and travel restrictions). In contrast, using the GBW dataset, we identified a substantial seasonal decline in the blackbird weekly reporting rate which was associated with a concomitant reduction in weekly count in gardens, but not in ecologically similar control species, which was contemporaneous with the period of detected USUV activity in Greater London. These population trends are consistent with a hypothesis of disease-mediated decline. Alternative explanations, such as variation in climate, food availability or bird movement need consideration and are discussed next.

Exploration of climate data indicates that, whilst the spring and early summer of 2020 was noteworthy with a high daily temperature average and low rainfall, at the time of USUV detection and the decline in the blackbird reporting rate, these parameters were within historical ranges (Supplementary Table 7). Consequently, while the climate may have been permissive for USUV transmission, there is no evidence to support variation in the weather alone as an explanation for the seasonal pattern of blackbird reporting rate decline; nor were declines observed in the robin or starling data, the control species with similar soil invertebrate diet and therefore similar vulnerability to summer drought. Blackbird, robin and starling populations in the UK are partially migratory; however, birds from mainland Europe do not migrate to overwinter in England until mid-October (i.e. after the decline in blackbird reporting rate occurred): consequently international bird movement does not offer an explanation for the observed regional blackbird decline. During the late summer season, short-distance movement from garden to non-garden habitats typically occurs, during the period of moult; however, the extent of the decline in blackbird reporting rate in gardens that occurred in Greater London in 2020 markedly exceeds that of the historical trend (2011–2019 inclusive; BTO *unpubl. data*). In summary, despite the fact that surveillance did not confirm further cases of wild bird USUV infection in 2020, and whilst it is not possible to ascribe causality, or exclude the chance that other factors may have contributed to the observed population trend, it remains possible that large-scale blackbird mortality due to USUV occurred in Greater London in summer 2020.

Our study and others^30^ illustrate the need to integrate disease surveillance and long-term population monitoring schemes to evaluate disease impact, and to use control species to explore potential confounding drivers of population change (e.g. climate, food availability). Since GBW reporting rates are generated online in real-time, and nationwide, they offer a tool to rapidly detect changes in species presence (i.e. reporting rate) or flock size in gardens (i.e. weekly maximum count) that can be used to strategically enhance surveillance effort for disease detection. As wild bird ring recovery reports are also submitted online, there is also the potential to develop a complementary system that monitors for trends in occurrence of dead birds that might signal a disease outbreak. The BBS survey provides the most robust available data on population trends to appraise disease impact, however there is a delay of some months until data from this scheme become available. Since repeated incursions have occurred in mainland Europe following first detection^1,17,31^, it is likely that USUV will emerge in the UK again, either through overwintering or repeat incursion(s). Integrated disease surveillance in combination with bird population monitoring using the various available datasets, as we have capitalised on here, is required to assess whether USUV re-occurs, or becomes endemic, in UK wild birds and to identify any associated population impacts.

By combining a range of professional and citizen science datasets our study approach facilitates the rapid detection of an emerging disease in free-living wildlife and enables insights into its incipient impact. We believe this multidisciplinary approach presents a framework for the early detection of disease outbreaks and incursion, thus helping to safeguard animal and public health. Such early warning systems could facilitate prompt mitigation action, for example targeted biosecurity measures and enhanced vigilance by medical and veterinary authorities. In addition, there is opportunity to further develop collaboration with ornithologists through active surveillance of wild birds, as was recently employed to detect West Nile virus in a migratory bird in the Netherlands^32^. Whilst population monitoring schemes are most developed for wild birds, lessons learned may be applied for the surveillance of diseases affecting other taxa.

## Supplementary Information

Supplementary Information 1.

Supplementary Information 2.

## Data Availability

All data generated or analysed during this study are included in this published article [and its supplementary information files].
