# Supplementary material for: Combining host and vector data informs emergence and potential impact of an Usutu virus outbreak in UK wild birds
Source: Sci Rep. 2022 Jun 18;12:10298. doi: 10.1038/s41598-022-13258-2 (PMC9206397; doi:10.1038/s41598-022-13258-2)
Supplement: Supplementary file 1 — Supplementary Information 1. [file 41598_2022_13258_MOESM1_ESM.docx]

**Supplementary files:**

*Lawson et al* *‘*Combining host and vector data informs emergence and potential impact of an Usutu virus outbreak in UK wild birds’

**Supplementary methods**

**1. Host surveillance at the index site**

Wild bird post-mortem examinations (PMEs) were conducted according to a standardised protocol ^1^. Microscopic examination of a saline mounted wet preparation of small intestinal tract contents was conducted to screen for evidence of protozoan or metazoan parasites. Liver, small intestinal tract contents, and gross lesions if present, were sampled for microbiological examination ^1^.

Tissue samples were fixed in 10% neutral buffered formalin and processed for histological examination, using routine methods, and stained with haematoxylin and eosin, plus Giemsa where indicated. Immunohistochemistry (IHC) was conducted as described in ^2^ for detection of flavivirus envelope (E) antigen on formalin-fixed, paraffin-embedded tissue sections from all six birds. Histological examination and appraisal of matched IHC slides were independently conducted by two veterinary pathologists: abnormalities were summarised, and qualitative consensus scores were assigned to the severity of histological lesions and intensity of envelope antigen staining.

DNA was extracted from pooled liver and spleen samples from each USUV-positive wild bird using a DNeasy Blood and Tissue extraction kit (Qiagen) and subjected to nested PCR for detection of *Plasmodium* and *Haemoproteus* spp., using a published protocol with minor modification ^3^. PCR products were run on 1.5% agarose gel, with amplicons of the anticipated length (524 base pair (bp)) extracted from the gel, purified using a QIAquick Gel Extraction Kit (Qiagen) and submitted for bidirectional Sanger sequencing. The National Centre for Biotechnology Institute (NCBI) Genbank and MalAvi database were searched to assign the lineage of any haemoparasites detected ^4^.

**2. Vector surveillance at the index site**

In summer 2015, as part of a vector monitoring programme, mosquitoes were collected using mouth and mechanical aspirators from resting sites in animal enclosures and surrounding vegetation at multiple locations across ZSL London Zoo. Specimens were preserved in 95% ethanol and species were identified based on morphology ^5,6^.

**2.1 Mosquito bloodmeal metabarcoding analysis in 2015**

Ninety-six blood-fed female *Culex pipiens* s.l. mosquitoes were processed individually with DNA extraction conducted as previously described. DNA extracts were amplified by PCR, using order-specific primers for birds and mammals targeting either the mitochondrially encoded ND2 (L5216 5’-GGC CCA TAC CCC TAA TG-3’ & H5766 5’-RGA KGA GAA RGC YAG GAT YTT KCK-3’) or Cytochrome Oxidase I (COIF 5’-TTC TCG AAC CAG AAA GAC ATT GGC AC-3’ & COIR 5’-ACT TCT GGG TGG CCA AAG AAT CAG AA-3’ or Vf1d 5′-TTC TCA ACC AAC CAC AAR GAY ATY GG-3′ & Vr1d 5′-TAG ACT TCT GGG TGG CCR AAR AAY CA-3′) genes ^7–9^. All PCR reactions were comprised of 4 μl 5x Green GoTaq Flexi Buffer, 2 μl 25 mM MgCl2, 0.1 μl GoTaq DNA Polymerase (Promega), 4 mM dNTPs (Bioline), 8.9 μl molecular grade water, 1 μl of each primer and 1 μl of gDNA. The PCR protocol for ND2 amplification employed an initial denaturation step at 94°C for 5 min, followed by 35 3‐step cycles consisting of 94°C for 30 s, 55°C for 1 min, and 72°C for 1 min, and a final extension at 72°C for 10 min. The cycling conditions for COIF/COIR comprised an initial denaturation step at 94°C for 5 min, followed by 40 cycles consisting of 94°C for 30 s, 55°C for 30 s, and 72°C for 1 min, and final extension at 72°C for 10 min. The cycling conditions for Vf1d/Vr1d comprised an initial denaturation step at 94°C for 5 min, followed by 35 cycles consisting of 94°C for 45 s, 51°C for 45 s, and 72°C for 1 min, and final extension at 72°C for 10 min.

PCR products were pooled by primer set and purified using the MinElute PCR purification kit (Qiagen). Cleaned amplicon pools were prepared for sequencing using the TruSeq Nano Library Preparation Kit (Illumina) following manufacturer’s protocols and using 100 ng of input material per library. All sequencing libraries were quantified using qPCR ^10^, normalized, and pooled in equimolar ratios prior to sequencing using 2 x 300 bp paired-end runs on an Illumina MiSeq platform at the Natural History Museum, London.

Initial sample demultiplex and adapter removal was performed using MiSeq Reporter Software. All metabarcoding datasets were curated and analysed using the DADA2 package in R ^11^. Due to the length of sequences targeted by the primers being greater than the combined R1 & R2 read lengths (650-700 bp) we processed just those reads containing the forward primer. Un-paired reads were filtered and trimmed using the following parameters; maximum number of ambiguous nucleotides = 0, minimum Phred quality score of 20, and trimmed to a sequence length of 200 bases based on visualisation of per-sample quality plots. In addition to this a hard trim was enforced from the 5’ end of each read to remove the primer sequence. Reads that did not meet these criteria following trimming were removed. As the TruSeq Nano library preparation can result in amplicons being sequenced from either direction, i.e. ligation of adapter sequences is not directional, the collapseNoMismatch function was used to align and collapse identical Amplicon Sequence Variants (ASVs). This step also merged sequences that differed in length but were otherwise identical. The most abundant sequence, irrespective of length, was taken forward as a representative of each collapsed ASV. The remaining ASVs were identified using the BLAST+ package ^12^ to compare sequences against NCBI nucleotide database (accessed 09.07.2021). Subsequently the Taxonomizr package in R was used to recover taxonomic information from NCBI accession numbers for each ASV.

**2.2 *Plasmodium* PCR and sequencing in 2015**

Parasite DNA was amplified by PCR, from the same samples as for bloodmeal metabarcoding, using the primer pair HAEMF (5′-ATG GTG CTT TCG ATA TAT GCA TG-3′) and HAEMR2 (5′-GCA TTA TCT GGA TGT GAT AAT GGT-3′), which are able to amplify various avian haemoparasite spp. ^13^. The PCR protocol employed an initial denaturation step at 95°C for 3 min, followed by 35 3‐step cycles consisting of 94°C for 30 s, 50°C for 30 s, and 72°C for 45 s, and a final extension at 72°C for 10 min. Reactions comprised of the same reagents as previously described for host specific amplicons. Amplicons of the anticipated length (480 bp) were visualised on a 1.5% agarose gel and submitted for bidirectional Sanger sequencing. For each sample that was positive for *Plasmodium*, individual PCRs were amplified and Sanger sequenced using the same primers as for metabarcoding, to identify hosts and potential reservoirs of infection. Amplicons generated by Sanger sequencing were assembled and manually checked/edited in Geneious R8 (Biomatters) prior to be being identified using the BLAST webserver (<https://blast.ncbi.nlm.nih.gov/Blast.cgi>).

**2.3 Mosquito monitoring and molecular surveillance for Usutu virus in 2020**

In response to the detection of USUV in wild birds, intensive mosquito trapping was implemented at the site during 266 trap nights over 26 nights during September 2020. Traps were located at four locations to assess the extent of mammalophagic versus ornithophagic mosquito host selection as well as to collect adult mosquitoes for molecular analysis (Supplementary Table 2). Two sites were situated next to outdoor avian exhibits (designated here as the African Bird Safari and Penguins) and the third adjacent to an outdoor mixed species exhibit (designated as the Mappin Pavilion); water bodies were in the vicinity of each of these three trapping sites. The fourth trapping site was adjacent to an office building and the outdoor animal areas of the veterinary hospital. Four different traps were employed which comprised two Mosquito Magnet® Executive Mosquito traps (MosquitoMagnet), five BG-Mosquitaire® (Biogents), three BG-Sentinel® (Biogents) and eight ovitraps. All BG traps were baited with BG-Lure, but no CO_2_  so should attract ornithophagic species. The Mosquito Magnet produced CO_2_, heat, and were baited with octenol lure so should attract mammalophagic species. Any resting mosquitoes found were also collected using an aspirator. Ovitraps, specific to detect non-native mosquito species, were constructed using black plastic pots (Ramona, 11 cm in width, 9 cm in height; Luwasa®, Interhydro AG), half filled with water and a polystyrene block (5x5x5 cm) provided for oviposition. Mosquitoes were collected every three days and kept in a cold chain at -20 ^o^C. Mosquito species identification was performed as above ^6^. *Culex pipiens* s.l. specimens were identified and females that had no evidence of a recent blood-feed were separated into pools of ten mosquitoes. RNA was extracted from each pool following homogenisation using a Qiagen RNA extraction kit (Qiagen) according to the manufacturer’s instructions. RNA was also extracted from individual *Culiseta annulata* mosquitoes using the same method. Samples were screened using a USUV specific RT-PCR ^14^. Positive RNA samples were submitted for NGS using the library preparation and sequencing as described in ^2^. We then used the UK USUV genome (GenBank accession number: MW001216) as a scaffold to map reads to and the resulting consensus sequence was visualised in Tablet v1.19.09.03.

Vero E6 cells grown in Dulbecco’s modified Eagle’s medium with 10% foetal calf serum were used to attempt virus isolation. An aliquot (100 mL) of each pooled mosquito homogenate that tested positive for USUV by RT-PCR was mixed with cells in suspension and transferred to a T25 flask. Cells were then incubated at 37^o^C with 5% CO_2_. Flasks were checked daily, using a phase-contrast microscope, for five days for any evidence of cytopathic effect. A second round of attempted virus isolation in Vero E6 cells was conducted using the same protocol. Total RNA extraction was conducted on cells from the second round and screened for USUV RNA using the PCR protocol ^15^.

In addition, DNA was extracted from engorged blood-fed mosquito abdomens following the protocols of ^16^ . Vertebrate host species in the blood meal were identified using a vertebrate-specific, M13-tailed, triple primer cocktail (VF1_t1 + VF1d_t1 + VF1i_t1 / VR1_t1 +VR1d_t1 + VD1i_t1) targeting a 685 bp sequence of the CO1 gene. This primer combination was expected to amplify all vertebrate species DNA. PCR reaction mix and conditions together with gel preparation and sequencing reactions followed the protocols of ^17^. All sequences were quality checked and edited using Lasergene version 12.1 (DNASTAR) and assigned to a particular vertebrate species when agreement was ≥98 % with sequences of named species in GenBank.

**3. Host investigations at the national scale**

**3.1 Molecular surveillance for Usutu virus in wild birds**

Scanning disease surveillance for wild birds, including Passeriformes and Strigiformes, is conducted in Great Britain through PMEs performed at both the Institute of Zoology (IoZ; [www.gardenwildlifehealth.org](http://www.gardenwildlifehealth.org)) and the network of Animal & Plant Health Agency (APHA) regional laboratories during the mosquito active season (April to November inclusive). Brain and kidney samples are routinely collected and submitted to APHA Weybridge for RNA extraction as described in ^2^. Samples from target species comprising Passeriformes (primarily blackbird used as a sentinel species) and Strigiformes are subjected to USUV RT-PCR analysis ^14^. Since 2015, USUV RT-PCR assays are also undertaken at APHA Weybridge on brain and kidney samples collected from Strigiformes species examined by the Predatory Bird Monitoring Scheme (PBMS; [www.pbms.ceh.ac.uk](http://www.pbms.ceh.ac.uk)).

Given the detection of USUV in wild birds for the first time in the UK in summer 2020 ^2^, samples from all garden bird and Strigiform submissions in 2020 to IoZ, APHA and the PBMS were subjected to specific USUV RT-PCR assay. Samples from captive birds that were examined post mortem at ZSL London Zoo over the period February-November 2020 inclusive were similarly tested for USUV; PMEs are routinely conducted on all captive and wild animals that die or are euthanased at ZSL London Zoo to inform understanding of collection health.

**3.2** **Syndromic surveillance and utilising the ring recovery dataset**

Reports of morbidity and mortality in garden wildlife (including birds) are solicited from members of the public and submitted online to the Garden Wildlife Health project ([www.gardenwildlifehealth.org](http://www.gardenwildlifehealth.org)), a citizen science scheme for wildlife disease surveillance. Disease incident reports (DIRs) were retrospectively evaluated over the available eight-year dataset, 2013-2020. The number of garden bird DIRs was compared with those for hedgehogs as a control group to account for variation in observer effort. Where observations of morbidity were available, garden bird DIRs were allocated to one of seven syndromic surveillance categories based on the clinical signs and circumstances of finding described by the reporter, combined with veterinary review of digital images when available. These comprised avian pox; beak/plumage abnormality; generalised ill health (e.g. lethargy, fluffed-up plumage); musculoskeletal disease; nestling mortality; neurological disease and predation/trauma. Morbidity reports which were not assigned to the above categories were grouped as ‘other’. DIRs with mortality for which no signs of morbidity were observed prior to death were categorised depending on the number of birds found dead (i.e. single or multiple mortality) over the same period. Based on the experience of wild bird mortality caused by USUV in mainland Europe (e.g. ^18^), the syndromic surveillance categories generalised ill health and neurological disease were considered of particular interest as a potential signal of infection. However, USUV could not be excluded as a possible underlying cause for DIRs categorised as musculoskeletal disease, trauma, predation, drowning or tick parasitism, or where blackbirds were found dead with no observed clinical signs.

The composition of syndromic surveillance categories for blackbird DIRs was compared across study years over the period June-November inclusive. This period was selected from the start of summer to the end of the active mosquito season in southern Britain given typical climate conditions. Chi-squared (χ^2^) tests were used to detect significant variations in the number of sites from which blackbird DIRs pertaining to each surveillance category were submitted in 2020, compared to the same period 2013-2019 inclusive, and with 2019 alone.

SaTScan ([www.satscan.org](http://www.satscan.org)) was used to conduct spatial clustering analysis of these categories of blackbird DIRs over the period June-November inclusive for each year investigated. Reports of avian pox, beak and plumage abnormality and nestling mortality were excluded from the spatial analyses since these conditions are not considered to be consistent with USUV infection. Briefly, the Kulldorff spatial scan statistic used in SaTScan compares the number of cases within a given area to the expected number of cases for that area, based on assumptions of random (i.e. Poisson distributed) occurrence and homogeneous individual risk ^19,20^. Likelihood ratio tests assess whether there is an increased risk of incident occurrence within a given cluster and a p-value is calculated for each cluster using Monte Carlo methods with 999 replications.

To account for spatial variation in observer effort, the number of sites with DIRs by county was analysed against a background population of the total number of sites per county from which passerine DIRs were submitted each year. To account for heterogeneity in blackbird population density, analysis was repeated using mean density (2007-2009) for this species derived from the BBS dataset as the background population (BTO *unpubl. data*).

We similarly categorised DIRs of house sparrow, the second species in which USUV infection was detected, and robin (*Erithacus rubecula*) and starling (*Sturnus vulgaris*), which were used as control species. Robin and starling are common passerine species with a widespread distribution across the UK whose diet primarily comprises ground and soil-living invertebrates (e.g. earthworms) commonly occurring in gardens ^21^, thus we expect would be affected by climate variation and food availability in a similar way to the blackbird but be unlikely to demonstrate USUV-mediated population decline. USUV-infection has been reported infrequently in these species to date, but concurrent virus detection in has always been much greater in blackbirds ^22–24^. Therefore, in the absence of challenge studies, available data support the likelihood of marked differential species susceptibility to this viral disease with blackbirds more sensitive compared with these two control species.

Volunteers participating in the British and Irish ringing scheme individually mark around 1 million birds per year (circa 25,000 blackbirds), of which circa 50,000 (circa 400 blackbirds) are subsequently found dead ^25^. Monitoring the number of recovered dead ringed wild birds offers an independent means to assess spatiotemporal patterns of wild bird mortality. We summarised reports of blackbirds ringed in any previous year and reported as found freshly dead (from any cause) by month of reporting for the periods 2013-2019 and 2020. We did this for all recoveries in Britain and Ireland, and also separately for Greater London, where the index site was located, and the NUTS2 (Nomenclature of Territorial Units for Statistics) regions of South East England and East of England, which have the closest proximity to mainland Europe.

**3.3 Sentinel species population monitoring**

Annual population changes of birds in the UK have been monitored through the BBS survey since 1994 using line transect surveys of a stratified random selection of 1 km squares; results are summarised nationally and by NUTS2 regions ^26^. The trends of BBS data for blackbirds since 2011 were compared for the UK, Greater London, South East England and East of England, to assess whether there was any evidence of population level decline over the period when incursion from mainland Europe is most likely to have occurred.

The number of birds visiting a sample of gardens nationally is recorded by volunteer participants in the GBW scheme ^21^. Gardens are self-selected and participants record the presence of each species in the garden and the maximum number observed at one time (i.e. proxy for flock size), on a weekly basis throughout the calendar year. We calculated the mean weekly reporting rate (i.e. proportion of gardens that submitted a record in that week in which a species was reported) for the period 2011-2019 for blackbird and house sparrow, the two species in which USUV was detected in the UK, and robin and starling as control species. As before, we calculated these rates separately for the UK as a whole, Greater London, South East England and East of England. Finally, we summarised the weekly maximum blackbird counts over the period 2003-2020 inclusive for Greater London alone.

**References**

1. Lawson, B. *et al.* Epidemiology of salmonellosis in garden birds in England and Wales, 1993 to 2003. *EcoHealth* **7**, 294–306 (2010).

2. Folly, A. J. *et al.* Detection of Usutu virus infection in wild birds in the United Kingdom, 2020. *Eurosurveillance* **25**, 2001732 (2020).

3. Waldenström, J., Bensch, S., Hasselquist, D. & Östman, Ö. A new nested polymerase chain reaction method very efficient in detecting *Plasmodium* and *Haemoproteus* infections from avian blood. *J. Parasitol.* **90**, 191–194 (2004).

4. Bensch, S., Hellgren, O. & Pérez‐Tris, J. MalAvi: a public database of malaria parasites and related haemosporidians in avian hosts based on mitochondrial cytochrome b lineages. *Mol. Ecol. Res.* **9**, 1353–1358 (2009).

5. Becker, N. *et al.* *Mosquitoes and Their Control*. (Springer-Verlag, 2010). doi:10.1007/978-3-540-92874-4.

6. Cranston, P. S. & etc. *Keys to the Adults, Male Hypopygia, Four-Instar Larvae and Pupae of the British Mosquitoes (Culicidae): With Notes on Their Ecology and Medical Importance*. (Freshwater Biological Assn., 1987).

7. Sorenson, M. D., Ast, J. C., Dimcheff, D. E., Yuri, T. & Mindell, D. P. Primers for a PCR-Based approach to mitochondrial genome sequencing in birds and other vertebrates. *Mol. Phylogenet. Evol.* **12**, 105–114 (1999).

8. Dove, C. J., Rotzei, N. C., Heacker, M. & Weigt, L. A. Using DNA barcodes to identify bird species involved in birdstrikes. *J. Wildl. Management* **72**, 1231–1236 (2008).

9. Ivanova, N. V., Dewaard, J. R. & Hebert, P. D. N. An inexpensive, automation-friendly protocol for recovering high-quality DNA: TECHNICAL NOTE. *Mol. Ecol. Notes* **6**, 998–1002 (2006).

10. High-Throughput Sequencing of Complete Mitochondrial Genomes | Springer Nature Experiments. https://experiments.springernature.com/articles/10.1007/978-1-4939-3774-5_3.

11. Callahan, B. J. *et al.* DADA2: High-resolution sample inference from Illumina amplicon data. *Nat. Methods.* **13**, 581–583 (2016).

12. Camacho, C. *et al.* BLAST+: architecture and applications. *BMC Bioinformatics* **10**, 421 (2009).

13. Hellgren, O., Waldenström, J. & Bensch, S. A new PCR assay for simultaneous studies of *Leucocytozoon, Plasmodium,* and *Haemoproteus* from avian blood. *J. Parasitol.* **90**, 797–802 (2004).

14. Jöst, H. *et al.* Isolation of Usutu Virus in Germany. *Am. J. Trop. Med. Hyg.* **85**, 551–553 (2011).

15. Johnson, N. *et al.* Assessment of a novel real-time pan-flavivirus RT-Polymerase chain reaction. *Vector-Borne and Zoonotic Di.* **10**, 665–671 (2010).

16. Brugman, V. A. *et al.* Molecular species identification, host preference and detection of myxoma virus in the *Anopheles maculipennis* complex (Diptera: Culicidae) in southern England, UK. *Parasit. Vectors* **8**, (2015).

17. Hernández-Triana, L. M. *et al.* Molecular approaches for blood meal analysis and species identification of mosquitoes (Insecta: Diptera: Culicidae) in rural locations in southern England, United Kingdom. *Zootaxa* **4250**, 67–76 (2017).

18. Steinmetz, H. W. *et al.* Emergence and establishment of Usutu virus infection in wild and captive avian species in and around Zurich, Switzerland—Genomic and pathologic comparison to other central European outbreaks. *Vet. Microbiol.* **148**, 207–212 (2011).

19. Kulldorff, M. A spatial scan statistic. *Communications in Statistics - Theory and Methods* **26**, 1481–1496 (1997).

20. Kulldorff, M., Huang, L. & Konty, K. A scan statistic for continuous data based on the normal probability model. *International Journal of Health Geographics* **8**, 58 (2009).

21. Cannon, A. R., Chamberlain, D. E., Toms, M. P., Hatchwell, B. J. & Gaston, K. J. Trends in the use of private gardens by wild birds in Great Britain 1995–2002. *J. Appl. Ecol.* **42**, 659–671 (2005).

22. Becker, N. *et al.* Epizootic emergence of Usutu virus in wild and captive birds in Germany. *PLoS One* **7**, e32604 (2012).

23. Chvala, S. *et al.* Monitoring of Usutu virus activity and spread by using dead bird surveillance in Austria, 2003-2005. *Vet. Microbiol.* **122**, 237–245 (2007).

24. Michel, F. *et al.* Evidence for West Nile Virus and Usutu Virus Infections in Wild and Resident Birds in Germany, 2017 and 2018. *Viruses* **11**, (2019).

25. Walker, R. H. *et al.* Bird ringing and nest recording in Britain and Ireland in 2017. *Ringing & Migration* **33**, 99–145 (2018).

26. Harris, S.J., Massimino, D., Balmer, D.E., Eaton, M.A., Noble, D.G., Pearce-Higgins, J.W., Woodcock, P. & & Gillings, S. The Breeding Bird Survey 2019. (2020).

27. Clé, M. *et al.* Usutu virus: A new threat? *Epidemiol. Infect.* **147**, (2019).

28. Vilibic-Cavlek, T. *et al.* Epidemiology of Usutu Virus: The European Scenario. *Pathogens* **9**, 699 (2020).

29. Garigliany, M.-M. *et al.* Detection of Usutu virus in a bullfinch (*Pyrrhula pyrrhula*) and a great spotted woodpecker *(Dendrocopos major*) in north-west Europe. *The Veterinary Journal* **199**, 191–193 (2014).

30. Lecollinet, S. *et al.* Dual emergence of Usutu Virus in Common Blackbirds, Eastern France, 2015. *Emerg. Inf. Dis.* **22**(12), (2016).

31. Rijks, J. *et al.* Widespread Usutu virus outbreak in birds in the Netherlands, 2016. *Euro Surveill.* **21**, (2016).

**Supplementary Materials 1.**

The preventative health protocol adopted at ZSL London Zoo following detection of USUV in synanthropic wild birds comprised:

1. Birds housed in the collection were reviewed to identify any passeriform or strigiform species considered at particular high risk of USUV-associated disease, based on reported mortality in mainland European zoological collections ^27^. Whilst no high risk species were kept at ZSL London Zoo, potential strategies that might have been applied during the mosquito active season include indoor housing and/or ectoparasitic treatment and/or enhanced mosquito vector control measures.
2. A site review of water bodies was conducted and actions were taken to reduce, cover or remove unnecessary standing water bodies for vector control.
3. Birdkeepers and clinical veterinary staff were encouraged to be vigilant for signs of morbidity or mortality in at risk bird species.
4. Routine RT-PCR screening of samples from collection birds that died during summer-autumn 2020 was conducted and detected no evidence of USUV infection or associated-mortality.
5. Enhanced mosquito monitoring and molecular screening for USUV was instigated to assess circulating virus and the extent of risk to captive bird health.

**Supplementary Figures**

**Supplementary Figure 1:**

1 2 3 4 5 6 7 8 9 10 11 12 13 14 15 16 17 18 19 20

300 bp

100 bp

**Supplementary Figure 1**. Agarose gel showing PCR-positive products. Detection of USUV in mosquito pools. ^14^ separation on a 1.5% agarose gel / 2-17 selected mosquito pools (6 pool #5; 8 pool #7; 12 pool #14; 13 pool #16; 16 pool #24; 17 pool #32), 18 no template control, 19 positive control, 1 & 20 gel pilot markers.

**Supplementary Figure 2.**

**
Supplementary Figure 2**. Number of Eurasian blackbird (*Turdus merula*, black) and robin (*Erithacus rubecula*, white) disease incident reports, June-November inclusive, 2013-2020 (left axis). For comparison, the number of hedgehog (*Erinaceus europaeus*, light grey; N=970) and total passerine (dark grey; N=7,665) disease incident reports are also presented (right axis). Note bars are offset for clarity.

**Supplementary Figure 3a.**

**House sparrow (*Passer domesticus*)**

| Suspect diagnosis | Number of DIR sites Jun-Nov | |
| --- | --- | --- |
|  | **2019** | **2020** |
| **Morbidity (+/- mortality)** |  |  |
| Avian pox | 11 | 15 |
| Found Dead - Multiple | 5 | 1 |
| Found Dead - Single | 14 | 31 |
| Generalised ill health | 20 | 42 |
| Musculoskeletal disease | 1 | - |
| Nestling Mortality | 1 | 12 |
| Neurological disease | 1 | 2 |
| Other | 9 | 4 |
| Plumage/Beak Abnormality | - | 4 |
| Predation/Trauma | 10 | 42 |
| **Mortality alone** |  |  |
| Found Dead - Multiple |  |  |
| Found Dead - Single |  |  |
| **Total** | **72** | **153** |


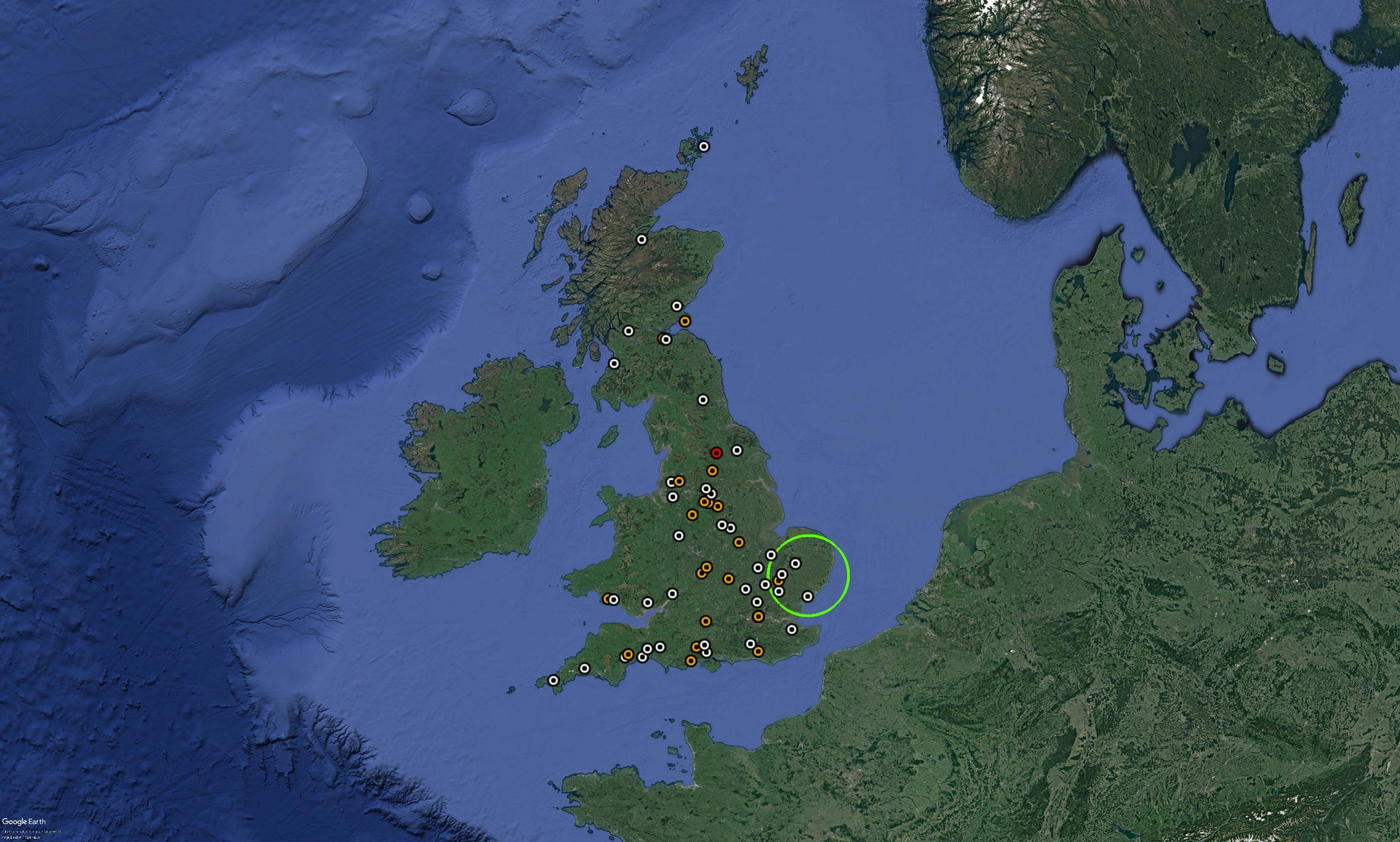


**A**

**B**


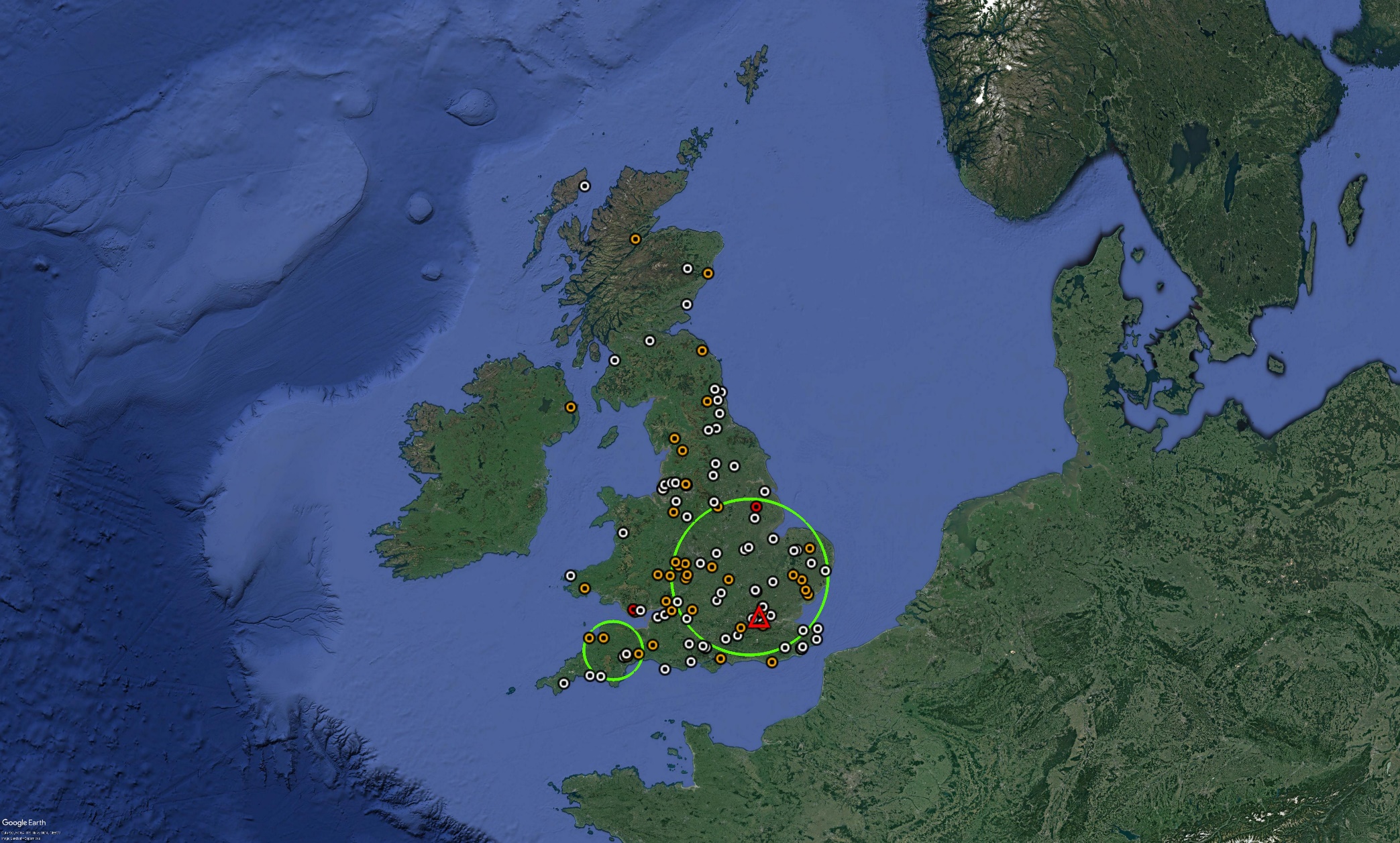


**Supplementary Figure 3a**. Distribution of disease incident reports (DIRs) of house sparrows (*Passer domesticus)*, June – November inclusive, 2019 (A; n = 60) and 2020 (B; n = 122). Red circles represent DIRs consistent with neurological disease; orange circles represent DIRs involving house sparrows exhibiting signs of generalised ill health; white circles represent all other morbidity and/or mortality DIRs (removing categories where USUV could reasonably be excluded as the cause i.e. avian pox, nestling mortality, beak and plumage abnormality); red triangle denotes site of confirmed USUV infection.

Green circles indicate statistically significant clusters of reports, accounting for spatial heterogeneity in house sparrow population density by using population indices from the British Trust for Ornithology Breeding Bird Survey (BTO, RSPB and JNCC, 2009) (A: LL = 10.2; RR = 9.3; p=0.004; B-south west: LL = 7.7; RR = 9.0; p = 0.04; B-east: LL = 12.7; RR = 2.7; p <0.001).

No statistically significant spatial clustering was detected for either year when analysed against a background population of number of sites from which passerine DIRs were submitted. Map created with Google Earth Pro. Version 7.3.3.7699 (2020) ([https://www.google.com/intl/en_uk/earth/versions/#earth-pro](https://eur03.safelinks.protection.outlook.com/?url=https%3A%2F%2Fwww.google.com%2Fintl%2Fen_uk%2Fearth%2Fversions%2F%23earth-pro&data=05%7C01%7Carran.folly%40apha.gov.uk%7Cca12ab01c8604cc3c16e08da3a25ef34%7C770a245002274c6290c74e38537f1102%7C0%7C0%7C637886231854771446%7CUnknown%7CTWFpbGZsb3d8eyJWIjoiMC4wLjAwMDAiLCJQIjoiV2luMzIiLCJBTiI6Ik1haWwiLCJXVCI6Mn0%3D%7C3000%7C%7C%7C&sdata=OlP1bjPTROSElkaowGVUkWygwyb6QCVEX%2BW6R5p%2FGZs%3D&reserved=0)).

**Supplementary Figure 3b.**

**Robin (*Erithacus rubecula*)**

| Suspect diagnosis | Number of DIR sites June-November inclusive | |
| --- | --- | --- |
|  | **2019** | **2020** |
| **Morbidity (+/- mortality)** |  |  |
| Avian pox | 5 | 6 |
| Beak/plumage abnormality | 2 | 10 |
| Generalised ill health | 3 | 9 |
| Musculoskeletal disease | 1 | - |
| Nestling Mortality | - | 1 |
| Neurological disease | - | 1 |
| Other | - | 3 |
| Predation/Trauma | 4 | 8 |
| **Mortality alone** |  |  |
| Found Dead - Multiple | - | 1 |
| Found Dead - Single | 4 | 11 |
| **Total** | **19** | **50** |


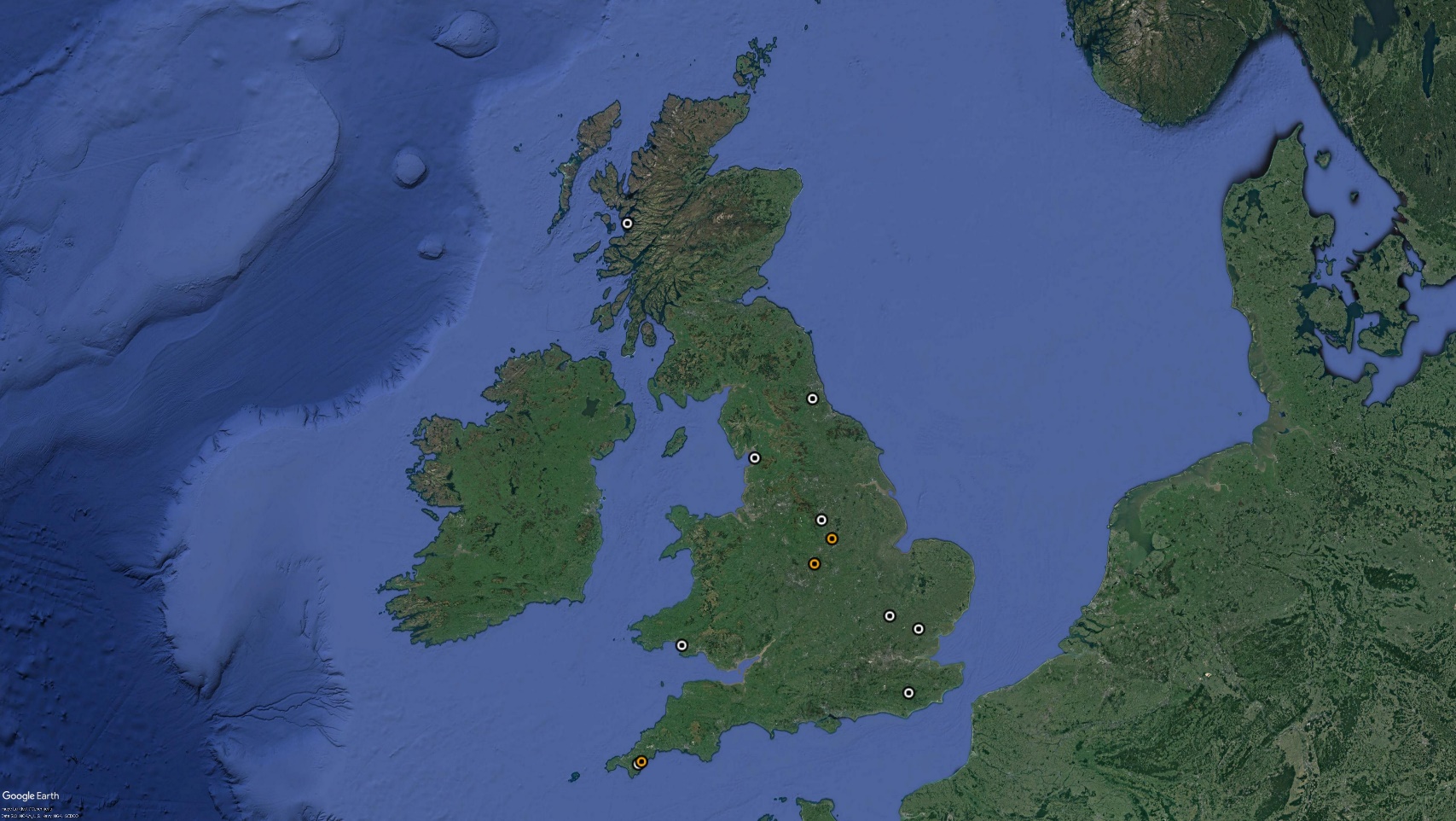


**A**


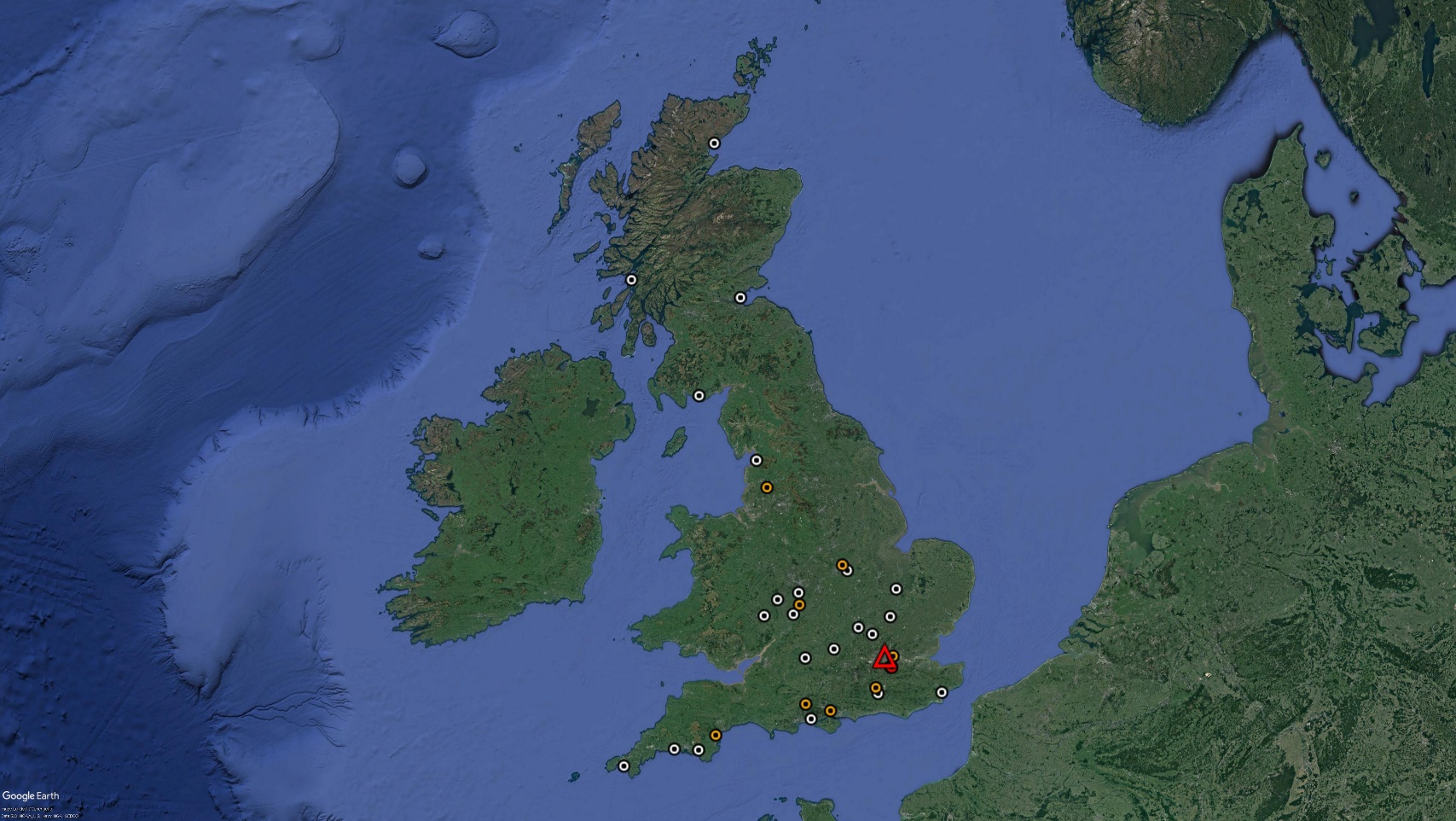


**B**

**Supplementary Figure 3b**. Distribution of disease incident reports (DIRs) of European robins (*Erithacus rubecula)*, June – November inclusive 2019 (A; n =12) and 2020 (B; n = 33). Red circles represent DIRs consistent with neurological disease; orange circles represent DIRs involving robins exhibiting signs of generalised ill health; white circles represent all other morbidity and/or mortality DIRs (removing categories where USUV could reasonably be excluded as the cause i.e. avian pox, nestling mortality, beak and plumage abnormality); red triangle denotes site of confirmed USUV infection. Map created with Google Earth Pro. Version 7.3.3.7699 (2020) ([https://www.google.com/intl/en_uk/earth/versions/#earth-pro](https://eur03.safelinks.protection.outlook.com/?url=https%3A%2F%2Fwww.google.com%2Fintl%2Fen_uk%2Fearth%2Fversions%2F%23earth-pro&data=05%7C01%7Carran.folly%40apha.gov.uk%7Cca12ab01c8604cc3c16e08da3a25ef34%7C770a245002274c6290c74e38537f1102%7C0%7C0%7C637886231854771446%7CUnknown%7CTWFpbGZsb3d8eyJWIjoiMC4wLjAwMDAiLCJQIjoiV2luMzIiLCJBTiI6Ik1haWwiLCJXVCI6Mn0%3D%7C3000%7C%7C%7C&sdata=OlP1bjPTROSElkaowGVUkWygwyb6QCVEX%2BW6R5p%2FGZs%3D&reserved=0)).

**Supplementary Figure 3c.**

**Starling (*Sturnus vulgaris)***

| Suspect Diagnosis | Number of DIR sites June-November inclusive | |
| --- | --- | --- |
|  | **2019** | **2020** |
| **Morbidity (+/- mortality)** |  |  |
| Avian pox | 3 | 3 |
| Beak/plumage abnormality | 0 | 0 |
| Generalised ill health | 6 | 7 |
| Musculoskeletal disease | 0 | 1 |
| Nestling Mortality | 0 | 2 |
| Neurological disease | 0 | 0 |
| Other | 1 | 0 |
| Predation/Trauma | 6 | 11 |
| **Mortality alone** |  |  |
| Found Dead - Multiple | 1 | 0 |
| Found Dead - Single | 6 | 13 |
| **Total** | **23** | **37** |

| 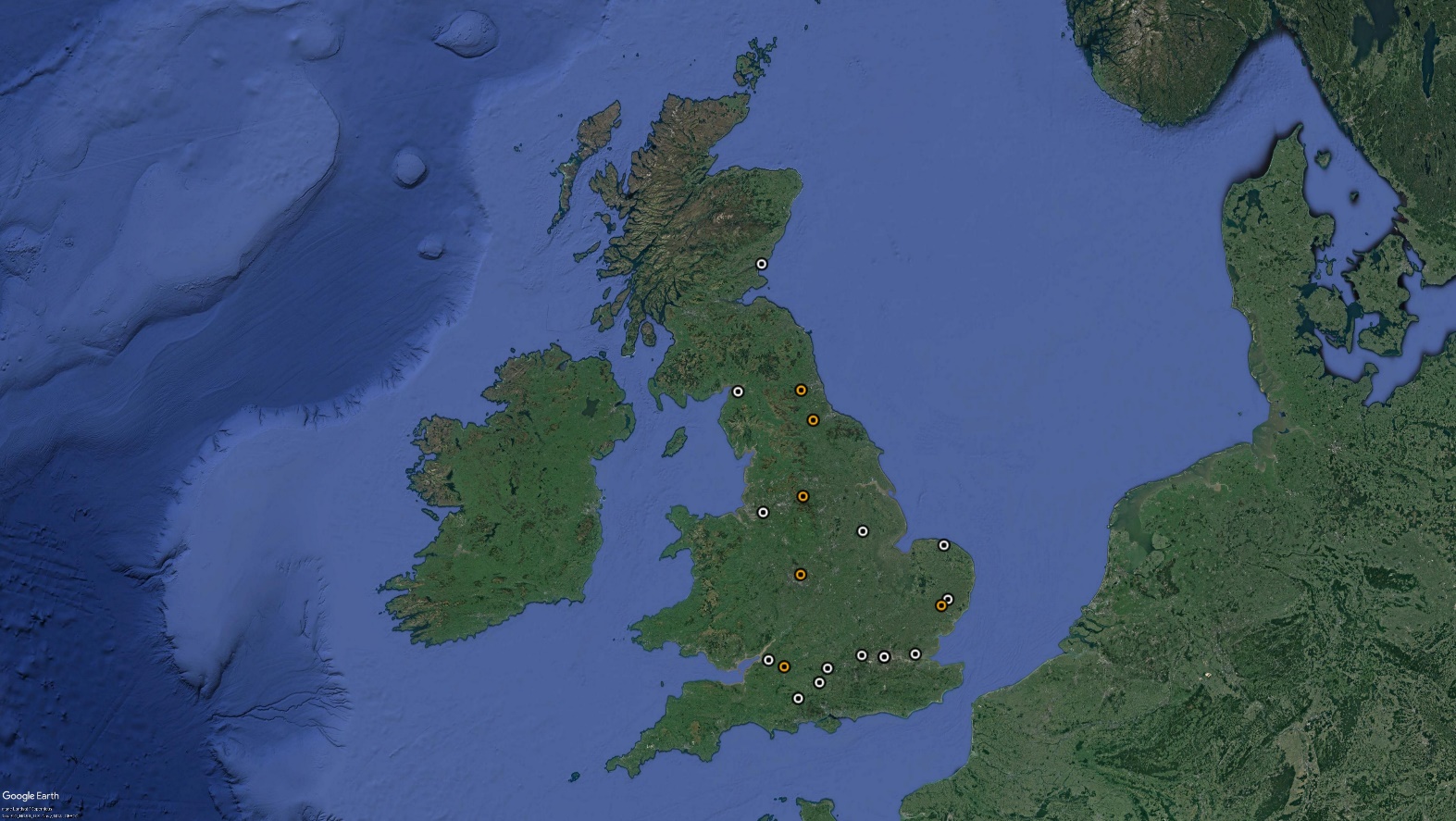  **A** | 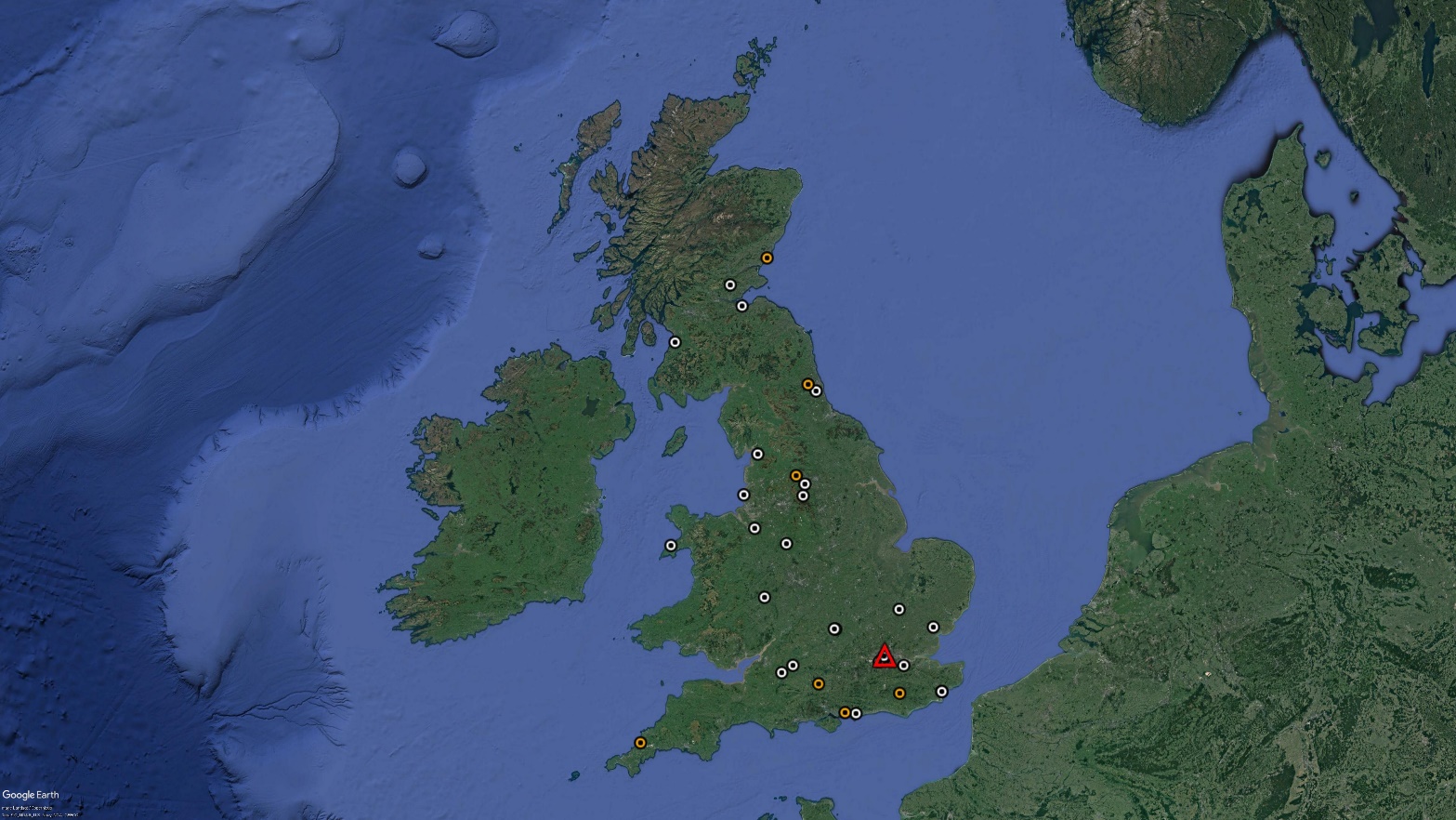  **B** |
| --- | --- |

**Supplementary Figure 3c**. Distribution of disease incident reports (DIRs) of common starling (*Sturnus vulgaris*), June – November inclusive, 2019 (A; n = 20) and 2020 (B; n = 32). Orange circles represent DIRs consistent with generalised ill health. White circles represent all other morbidity and/or mortality DIRs (removing categories where USUV could reasonably be excluded as the cause i.e. avian pox, nestling mortality, beak and plumage abnormality); red triangle denotes site of confirmed USUV infection. Map created with Google Earth Pro. Version 7.3.3.7699 (2020) ([https://www.google.com/intl/en_uk/earth/versions/#earth-pro](https://eur03.safelinks.protection.outlook.com/?url=https%3A%2F%2Fwww.google.com%2Fintl%2Fen_uk%2Fearth%2Fversions%2F%23earth-pro&data=05%7C01%7Carran.folly%40apha.gov.uk%7Cca12ab01c8604cc3c16e08da3a25ef34%7C770a245002274c6290c74e38537f1102%7C0%7C0%7C637886231854771446%7CUnknown%7CTWFpbGZsb3d8eyJWIjoiMC4wLjAwMDAiLCJQIjoiV2luMzIiLCJBTiI6Ik1haWwiLCJXVCI6Mn0%3D%7C3000%7C%7C%7C&sdata=OlP1bjPTROSElkaowGVUkWygwyb6QCVEX%2BW6R5p%2FGZs%3D&reserved=0)).

**Supplementary Figure 4.**

**Supplementary Figure 4**. Number of dead ringed Eurasian blackbird (*Turdus merula*) recovery reports in Britain and Ireland (n=287/year in 2013-2019; n=212 in 2020) (a); Greater London and South East England combined due to small sample size (n=37/year; 28) (b); and Eastern England (n=82/year; 55) (c). 2013-19 pink lines and 2020 green lines.

**Supplementary Figure 5.**

**Supplementary Figure 5**. Breeding Bird Survey population indices (relative to 1994 when the smoothed index is set to 100) for Eurasian blackbird (*Turdus merula*) for the UK, Greater London, South East and East of England regions (1995-2019 inclusive). A moving average trend line has been included for each of the geographic regions. BBS data were not available for 2020 as survey effort was substantially reduced due to COVID-19 related movement restrictions on surveyors.

The year when USUV-associated wild bird mortality was first detected in the coastal countries of Western Europe is marked on the figure ^28^: 2011 in Germany (DE)^22^; 2012 in Belgium (BE) ^29^; 2015 in France (FR) ^30^ and 2016 in The Netherlands (NL) ^31^. Incursion to the UK and any associated large-scale blackbird mortality sufficient to cause disease-mediated population decline would be predicted to occur subsequent to first detection in these countries based on spatio-temporal patterns of USUV spread in mainland Europe.

**Supplementary Figure 6**


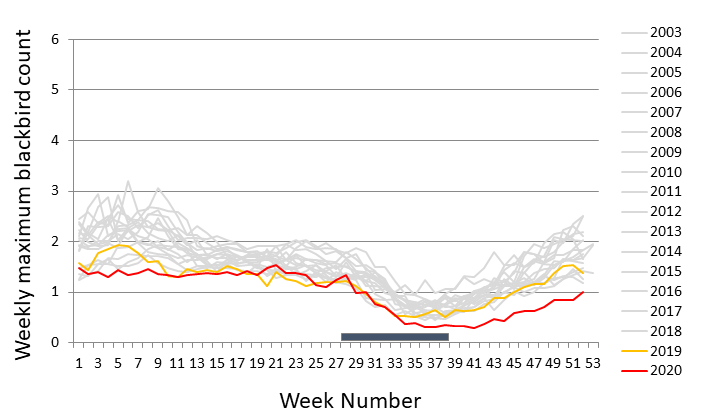


**Supplementary Figure 6**: Weekly maximum Eurasian blackbird (*Turdus merula*) counts in gardens from the BTO Garden BirdWatch data, 2003-2020 inclusive for Greater London. The grey horizontal line represents the period from first USUV detection (15^th^ July 2020) through blackbird post-mortem examination and latest detection (17^th^ September 2020) through mosquito surveillance and molecular testing.

**Supplementary Tables**

**Supplementary Table 1**: Post-mortem examination and ancillary diagnostic findings of Usutu virus RT-PCR-positive wild birds GB=Gall bladder; GIT=Gastrointestinal tract; NAD = No abnormalities detected; NSF=No significant findings; SI= Small intestine

| **Species & unique Identifier** | **Date dead** | **History** | **Signalment & body condition & BW (g)** | **Carcass state** | **Key macroscopic findings** | **Microbiology** | **Parasitology** | **Histopathology** | **Usutu virus RT-PCR Cycle threshold (Ct) value for pooled brain and kidney** |
| --- | --- | --- | --- | --- | --- | --- | --- | --- | --- |
| Blackbird  1 | 15/07/20 | Dehydrated, not gripping, euthanased | Adult male  Thin  84.3 g | Fresh  Mild autolysis | Urates in ureters (suspect dehydration)  GIT normal volume contents  Acanthocephalans and cestodes in SI (likely incidental)  **Liver:** NAD  **Spleen:** NAD | Liver: NSF  SI contents: *Campylobacter* sp. | Faeces wet prep:  Negative | Examined: Brain, heart, kidney, proventriculus/gizzard, SI, testis, parasite and syrinx  **Heart**: Minimal, multifocal, lymphoplasmacytic myocarditis  **Kidney**: Minimal, multifocal, lymphoplasmacytic, interstitial nephritis  **Proventriculus:** Minimal, focal, lymphoplasmacytic mural proventriculitis | 31.03 |
| Blackbird  2 | 19/07/20 | Found dead | Adult male  Emaciated  73.0 g | Fresh  Moderate autolysis | Urate staining around vent  Skin adherent to underlying musculature (suspect dehydration)  Mild serosal vessel congestion of proximal SI  Scant GIT contents  **Liver**: Dark red, mild hepatomegaly (4.9 g)  **Spleen**: dark red, splenomegaly (38.8 x 6.1 x 6.1 mm; 0.9 g) | Liver: NSF  SI contents: NSF | SI contents wet prep:  Negative | Examined: Brain, duodenum, fat, heart, kidney, liver, lung, pancreas, trachea and skeletal muscle, spleen, vagus nerve  **Fat**: Serous atrophy with mild, multifocal, lymphoplasmacytic and granulomatous steatitis  **Kidney:** Marked, diffuse sloughing of arterial endothelial cells  **Liver**: Moderate, spotty, portal to random, lymphoplasmacytic and necrotising hepatitis  **Pancreas:** Mild, multifocal, pancreatic necrosis  **Spleen**: Moderate, multifocal, plasmacytic and necrotising splentitis  **Vagus nerve:** Moderate, focal, lymphoplasmacytic neuritis | 29.98 |
| Blackbird  3 | 15/07/20 | Found dead | Adult male  Emaciated  71.0 g | Frozen  Moderate autolysis | Skin adherent to underlying musculature (suspect dehydration)  Bilateral femoral fractures – no associated haemorrhage, suspect scavenger damage  GIT scant to moderate contents  **Liver**: NAD (2.5 g)  GB: moderate volume normal bile material with 3x small (~1 mm length), black metazoan parasites (suspect trematodes, likely incidental)  **Spleen**: No evidence of generalised enlargement but elongated profile, likely NAD (47 x 3 x 3 mm; 0.1 g) | Liver: NSF  SI contents: NSF | SI contents wet prep: Negative | Examined: Brain, bursa of Fabricius, heart, kidney, liver, lung spleen, skeletal muscle, trachea  **Brain:** Small focus of necrotic debris in leptomeninges  **Liver**: Diffuse, moderate, haemosiderosis  **Skeletal muscle:** Minimal, multifocal, lymphoplasmacytic myositis  **Vagus nerve:** Moderate, focal, lymphoplasmacytic neuritis | 23.42 |
| Blackbird  4 | 27/07/20 | Found unresponsive and immobile, died | Juvenile undetermined sex  Normal condition  70.9 g | Fresh  Mild autolysis | Muscle pallor (suspect anaemia) Prominent subcutaneous vessels on bilateral flanks, epicardial and serosal GIT vessels. Skin adherent to underlying musculature (suspect dehydration) GIT scant to moderate contents Heavy mixed burden of metazoan intestinal parasites. **Liver**: Dark plum colour, possible hepatomegaly (4.54 g) **Spleen**: plum-coloured, prominent size/equivocal splenomegaly  (24 x 4 x 4 mm; 0.47 g) | Liver: NSF  SI contents: NSF | SI contents wet prep: cestodes and ascarids  *Plasmodium matutinum* LINN1  GenBank: [MT912164.1](https://www.ncbi.nlm.nih.gov/nucleotide/MT912164.1?report=genbank&log$=nucltop&blast_rank=5&RID=BH5ACYYK013) | Examined: Brain, bursa of Fabricius, heart, GIT, kidney, liver, lung, skeletal muscle, spleen, trachea  **Bursa**: Marked, diffuse, medullary lymphocytolysis  **Kidney**: Moderate, multifocal lymphoplasmacytic and histiocytic interstitial nephritis  **Liver**: Mild, spotty, portal to random lymphoplasmacytic and necrotising hepatitis with sinusoidal endothelial sloughing  **Pancreas**: Mild, multifocal pancreatic necrosis  **Skeletal muscle**: Mild, multifocal lymphoplasmacytic myositis  **Spleen**: Mild, multifocal peri-ellipsoidal necrosis | 23.20 |
| Blackbird  5 | 03/08/20 | Found dead | Juvenile undetermined sex  Normal condition  71.2 g | Fresh  Mild autolysis | Multiple skin and pectoral muscle wounds, fractures and internal haemorrhage consistent with predation  GIT normal volume contents  Mixed endoparasitism (SI cestode, acanthocephalan, ascarid nematode) and *Syngamus trachea*) (likely incidental) **Liver:** plum-coloured friable & fragmented (4.54 g)  **Spleen:** NLD (28 x 3 x 4 mm; 0.41 g) | Liver: NSF  SI contents: *Campylobacter* sp. | SI contents wet prep: negative  *Plasmodium vaughani* SYAT05  GenBank: [MT912207.1](https://www.ncbi.nlm.nih.gov/nucleotide/MT912207.1?report=genbank&log$=nucltop&blast_rank=4&RID=BH5G2587016) | Examined: Heart, GIT, kidney, liver, lung, pancreas, skeletal muscle, spleen, trachea  **Kidney**: Moderate multifocal lymphoplasmacytic, histiocytic and necrotising interstitial nephritis  **Liver**: Mild, spotty, periportal lymphoplasmacytic and necrotising hepatitis  **Lung:** Marked, diffuse, peracute, alveolar haemorrhage consistent with severe trauma  **Pancreas**: Mild, multifocal, pancreatic necrosis  **Skeletal muscle**: Mild, multifocal, lymphoplasmacytic myositis  **Spleen**: Mild, multifocal peri-ellipsoidal lymphoplasmacytic and necrotising splenitis | 24.84 |
| House sparrow | 26/08/20 | Found dead | Adult male  Thin  21.5 g | Fresh  Mild autolysis | Kidneys diffuse pale tan discolouration.  Gizzard empty but intestinal tract normal volume contents  **Liver**: NAD  **Spleen**: NAD | Liver: NSF  Faeces: NSF | Faeces wet prep: numerous coccidian oocysts | Examined: Brain, heart, kidney, liver, lung, oesophagus, pancreas, skeletal muscle  **Kidney:** Moderate, multifocal renal tubular necrosis with minimal, focal lymphoplasmacytic interstitial nephritis  **Liver:** Mild, diffuse, hepatocellular glycogenosis  **Oesophagus:** Focal necrosis of oesophageal gland | 24.62 |

**Supplementary Table 2.** Details of mosquito species and number caught across four trapping locations (266 trapping nights) at ZSL London Zoo, the index site of Usutu virus detection in Greater London, over the period 8^th^ September – 21^st^ September 2020.

| **Location** | **Trap type** | **Number of nights** |  | ***Cx. pipiens* s.l.** | | |  | ***Cs. annulata*** | | |  | **Total** |
| --- | --- | --- | --- | --- | --- | --- | --- | --- | --- | --- | --- | --- |
|  |  |  |  | ♀ | ♀/night | ♂ |  | ♀ | ♀/night | ♂ |  |  |
| Penguins | Magnet (P1) | 13 |  | 0 | 0 | 0 |  | 0 | 0 | 0 |  | 0 |
|  | Sentinel (P2) | 26 |  | 83 | 3.29 | 0 |  | 0 | 0 | 0 |  | 83 |
|  | Mosquitaire (P3) | 26 |  | 15 | 0.57 | 3 |  | 0 | 0 | 1 |  | 19 |
|  | Mosquitaire (P4) | 19 |  | 10 | 0.52 | 0 |  | 0 | 0 | 0 |  | 10 |
|  | Mosquitaire (P5) | 26 |  | 15 | 0.53 | 0 |  | 0 | 0 | 0 |  | 15 |
| African Bird Safari | Sentinel (A1) | 26 |  | 5 | 0.19 | 1 |  | 0 | 0 | 0 |  | 6 |
|  | Mosquitaire (A2) | 26 |  | 243 | 9.3 | 7 |  | 2 | 0.08 | 0 |  | 252 |
|  | Resting (AR)* | NA |  | 29 | NA | 4 |  | 2 | NA | 0 |  | 35 |
| Mappin Pavilion | Mosquitaire (M1) | 26 |  | 6 | 0.23 | 0 |  | 0 | 0 | 0 |  | 6 |
|  | Sentinel (M2) | 26 |  | 35 | 1.35 | 0 |  | 0 | 0 | 0 |  | 35 |
| Vet Dept | Magnet (V1) | 19 |  | 27 | 1.42 | 1 |  | 0 | 0 | 0 |  | 28 |
| TOTAL |  |  |  | 468 |  | 16 |  | 4 |  | 1 |  | 489 |

*manual collection using aspirator.
Footer: Eight ovitraps were deployed from which no eggs or larvae were recovered.

**Supplementary Table 3**. Collection date, cycle threshold value and number of reads mapped to the Greater London Usutu virus genome (Genbank Accession number MW001216) for each of the six *Cx. pipiens* s.l. pools collected in Greater London 2020, that tested positive by an Usutu virus specific RT-PCR. Ambiguous test results with high Ct values (cycle threshold >35) marked with an asterisk.

| Mosquito pool | Collection date | Usutu specific RT-PCR cycle threshold value | Number of reads mapped to the Greater London Usutu virus genome |
| --- | --- | --- | --- |
| #5 | 8-10^th^ September 2020 | 28.24 | 184 |
| #7 | 8-10^th^ September 2020 | 28.84 | 125 |
| #14* | 8-10^th^ September 2020 | 36.54 | 4 |
| #16 | 8-10^th^ September 2020 | 29.95 | 52 |
| #24 | 17^th^ September 2020 | 31.79 | 143 |
| #32* | 21^st^ September 2020 | 44.94 | 2 |

**Supplementary Table** **4**: Species breakdown of wild birds tested negative by RT-PCR for Usutu virus, 2012-2019 inclusive.
Asterisk denotes captive owls.

| **Order/Family** | **Species** | **Total** | **Submissions per annum** |
| --- | --- | --- | --- |
| **Passeriformes** |  |  |  |
| Corvidae | Carrion crow (*Corvus corone*) | 11 | 1-5 |
|  | Magpie (*Pica pica*) | 2 | 0-1 |
|  | Raven (*Corvus corax*) | 4 | 0-3 |
| Fringillidae | Chaffinch (*Fringilla coelebs*) | 2 | 0-2 |
| Turdidae | Eurasian blackbird (*Turdus merula*) | 43 | 2-8 |
|  | Fieldfare (*Turdus pilaris*) | 3 | 0-3 |
| Sturnidae | Starling (*Sturnus vulgaris*) | 3 | 0-3 |
| **Strigiformes** |  |  |  |
| Strigidae | Eagle owl (*Bubo bubo*)* | 2 | 0-1 |
|  | Little owl (*Athene noctua*) | 7 | 0-2 |
|  | Long-eared owl (*Asio otus*) | 2 | 0-1 |
|  | Short-eared owl (*Asio flammeus*) | 5 | 0-3 |
|  | Snowy owl (*Bubo scandiacus*)* | 2 | 0-1 |
|  | Tawny owl (*Strix aluco*) | 85 | 0-26 |
| Tytonidae | Barn owl (*Tyto alba*) | 201 | 0-51 |

**Supplementary Table 5:** Species composition of birds tested for Usutu virus by RT-PCR (a) Wild birds from across Great Britain, April-November inclusive 2020 and (b) Captive birds at ZSL London Zoo, February-November inclusive 2020

**(a)**

| **Order** | **Family** | **Species** | **Total  (No of sites)** | **Total PCR positive (No of sites)** |
| --- | --- | --- | --- | --- |
| **Accipitriformes** | Accipitridae | Eurasian sparrowhawk (Accipiter nisus) | 1(1) | 0 |
| **Apodiformes** | Apodidae | Common swift (*Apus apus*) | 1(1) | 0 |
| **Columbiformes** | Columbidae | Feral pigeon (*Columba livia*)* | 2(2) | 0 |
|  | Columbidae | Wood pigeon (*Columba palumbus*)* | 6 (2) | 0 |
| **Falconiformes** | Falconidae | Common kestrel (*Falco tinnunculus*) | 3(3) | 0 |
| **Passeriformes** | Fringillidae | Chaffinch (*Fringilla coelebs*) | 11(10) | 0 |
|  | Fringillidae | Goldfinch (*Carduelis carduelis*) | 2(2) | 0 |
|  | Fringillidae | Greenfinch (*Chloris chloris*) | 9(8) | 0 |
|  | Fringillidae | Twite (*Linaria flavirostris*) | 1(1) | 0 |
|  | Muscicapidae | Spotted flycatcher (*Muscicapa striata*) | 1(1) | 0 |
|  | Passeridae | House sparrow (*Passer domesticus*)^ø^ | 7 (4) | **1(1)** |
|  | Paridae | Blue tit (*Cyanistes caeruleus*) | 6(3) | 0 |
|  | Paridae | Coal tit (*Periparus ater)* | 1(1) | 0 |
|  | Paridae | Great tit (*Parus major*) | 1(1) | 0 |
|  | Prunellidae | Dunnock (*Prunella modularis*) | 1(1) | 0 |
|  | Phylloscopidae | Willow warbler (*Phylloscopus trochilus*) | 1(1) | 0 |
|  | Sturnidae | Starling (*Sturnus vulgaris*) | 1(1) | 0 |
|  | Sylviidae | Blackcap (*Sylvia atricapilla*) | 2(2) | 0 |
|  | Turdidae | Blackbird (*Turdus merula*)^#^ | 15 (10) | **5(1)** |
|  | Turdidae | Song thrush (*Turdus philomelos*) | 4(3) | 0 |
| **Psittaciformes** | Psittacidae | Rose-ringed parakeet (*Psittacula krameri*)* | 1(1) | 0 |
| **Strigiformes** | Strigidae | Tawny owl (*Strix aluco*)* | 2(2) | 0 |
|  | Tytonidae | Barn owl (*Tyto alba*) | 36(36) | 0 |
| **TOTAL** |  |  | 115 (90) | 6(1)) |

Footer: Fourteen wild birds were submitted by the APHA regional labs, 67 by IoZ and 34 by the PBMS. Wild birds were from 90 sites across Great Britain, comprising all nine English regions (80 sites) along with Scotland (four sites) and Wales (six sites).

*Mortality of an additional four wild bird species was detected at the index site over the five-month period July-November 2020, comprising feral pigeons (*Columba livia*), wood pigeons (*Columba palumbus*), a rose-ringed parakeet (*Psittacula krameri*) and a tawny owl (*Strix aluco*): no USUV infection was detected in these columbiform, psittaciform or strigiform species.
^#^ Blackbirds comprised submissions from Greater London (5/5 tested USUV PCR-positive; from index site), East of England (0/4; 4 sites), North East (0/2; 1 site); South West (0/1; 1 site), West Midlands (0/2; 2 sites) and Wales (0/1; 1 site).  ^ø^House sparrows comprised submissions from Greater London (1/4 tested USUV PCR positive; submitted from index site), East Midlands (0/1; 1 site), North East (0/1; 1 site) and South West (0/1; 1 site).

**Supplementary Table 5 (b)**

| **Order** | **Family** | **Species** | **Total** | **PCR positive** |
| --- | --- | --- | --- | --- |
| **Anseriformes** | Anatidae | Madagascar teal (*Anas bernieri)* | 1 | 0 |
| **Ciconiiformes** | Ciconiidae | Abdim's stork (*Ciconia abdimii)* | 1 | 0 |
| **Columbiformes** | Columbidae | Grey-capped emerald dove (*Chalcophaps indica)* | 2 | 0 |
|  | Columbidae | Superb fruit dove (*Ptilinopus superbus)* | 1 | 0 |
|  | Columbidae | Victoria crowned pigeon (*Goura victoria)* | 1 | 0 |
| **Passeriformes** | Estrildidae | Red-cheeked cordon-bleu (*Uraeginthus bengalus)* | 1 | 0 |
|  | Estrildidae | Java sparrow (*Lonchura oryzivora)* | 1 | 0 |
|  | Icteridae | Montserrat oriole (*Icterus oberii*) | 1 | 0 |
|  | Leiothrichidae | Blue-crowned laughing thrush (*Pterorhinus courtoisi)* | 2 | 0 |
|  | Ploceidae | Red bishop weaver (*Euplectes orix)* | 1 | 0 |
|  | Nectariniidae | Scarlet-chested songbird *(Chalcomitra senegalensis)* | 1 | 0 |
|  | Sturnidae | Bali myna (*Leucopsar rothschildi)* | 1 | 0 |
| [**Musophagiformes**](https://en.wikipedia.org/wiki/Turaco) | [Musophagidae](https://en.wikipedia.org/wiki/Turaco) | Violet turaco (*Musophaga violacea)* | 1 | 0 |
| **Pelecaniformes** | Scopidae | Hamerkop (*Scopus umbretta*) | 1 | 0 |
|  | Threskiornithidae | Waldrapp ibis *(Geronticus eremita)* | 1 | 0 |
| **Sphenisciformes** | Spheniscidae | Humboldt penguin *(Spheniscus humboldti)* | 2 | 0 |
| **TOTAL** |  |  | 19 | 0 |

Footer: The captive birds comprised 11 held in indoor aviaries, which included all seven Passeriformes; two in an outdoor only enclosure, and the remaining six birds with mixed outdoor and indoor housing.

**Supplementary Table 6**: Number of Eurasian blackbird (*Turdus merula*) disease incident reports submitted in 2013-2020 by syndromic surveillance category over the period June-November inclusive

| Year(s) | Avian pox | Beak/ plumage abnormality | Generalised ill health | Musculoskeletal disease | Neurological disease | Nestling mortality | Predation/ Trauma | Other | Total |
| --- | --- | --- | --- | --- | --- | --- | --- | --- | --- |
| 2013-2018 | 10 | 28 | 36 | 5 | 3 | 0 | 19 | 6 | 107 |
| 2019 | 8 | 13 | 5 | 2 | 1 | 1 | 20 | 6 | 56 |
| 2020 | 2 | 17 | 26 | 3 | 5 | 3 | 42 | 4 | 102 |
| Total | 20 | 58 | 67 | 10 | 9 | 4 | 81 | 16 | 265 |

**Supplementary Table 7:** Weather data from office.gov.uk/research/climate/maps-and-data/about/archives

| Region | Mean of daily maximum temperature (^o^C) (1884-2020 inclusive) | | Total precipitation amount (cm)  (1862-2020 inclusive) | |
| --- | --- | --- | --- | --- |
|  | Spring 2020 | Summer 2020 | Spring 2020 | Summer 2020 |
| England SE and Central (1884-2020 inclusive) | 15.57  (3^rd^ highest in rank order) | 22.03  (20^th^ in rank order) | 59.5  (2^nd^ lowest in rank order) | 260.8  (22^nd^ in rank order) |
| East Anglia | 15.34  (3^rd^ highest in rank order) | 22.21  (18^th^ in rank order) | 54.8  (5^th^ lowest in rank order) | 181.2  (60^th^ in rank order) |
